# Effect of administration of a probiotic preparation on gut microbiota and immune response in healthy women in India: an open-label, single-arm pilot study

**DOI:** 10.1186/s12876-018-0819-6

**Published:** 2018-06-15

**Authors:** Ankita Singh, Aditya N. Sarangi, Amit Goel, Rajni Srivastava, Rajat Bhargava, Priyanka Gaur, Amita Aggarwal, Rakesh Aggarwal

**Affiliations:** 1 Departments of Immunology, Sanjay Gandhi Postgraduate Institute of Medical Sciences, Lucknow, 226014 India; 2 Gastroenterology, Sanjay Gandhi Postgraduate Institute of Medical Sciences, Lucknow, 226014 India; 3 Biomedical Informatics Center, Sanjay Gandhi Postgraduate Institute of Medical Sciences, Lucknow, 226014 India

**Keywords:** Probiotic, Gut microbiome, Immune response, VSL#3, Healthy women

## Abstract

**Background:**

Probiotics have been shown to be useful for the treatment of many disease conditions. These beneficial effects are believed to be mediated by change in the composition of gut microbiota and modulation of the host immune responses. However, the available data on the effect of probiotics on these parameters are quite limited.

**Methods:**

We studied the composition of fecal microbiota, using 16S rRNA sequencing, and host immune responses in peripheral blood (plasma cytokine levels, T cell subsets and in vitro cytokine production after stimulation with anti-CD3/CD28 antibody or lipopolysaccharide) in a group of 14 healthy women at three time-points – before and after administration of a probiotic preparation (a capsule of VSL#3, each containing 112.5 billion freeze-dried bacterial cells belonging to 8 species, twice a day for 4 weeks), and 4-weeks after discontinuation of the probiotic administration.

**Results:**

There was no change in the abundance of various bacterial taxa as well as in the alpha diversity of gut microbiota following administration of the probiotic, or following its discontinuation. Probiotic administration led to a reduction in the relative frequency of circulating Th17 cells, and in vitro production of cytokines in whole-blood cultures in response to lipopolysaccharide stimulation. However, it had no effect on the relative frequencies of Th1, Th2 and T regulatory cells among circulating peripheral blood mononuclear cells, on plasma cytokine levels and on in vitro production of cytokines by T cells.

**Conclusions:**

We found that VSL#3 administration did not lead to any changes in gut flora, but led to a reduction in the frequency of Th17 cells and in the production of pro-inflammatory cytokine on lipopolysaccharide stimulation. These findings suggest that the beneficial anti-inflammatory effect of this preparation in patients with autoimmune and allergic disorders may be related to reduced production of monocyte-derived cytokines rather than to changes in the composition of gut microbiota.

**Trial registration:**

NCT03330678, Date of registration 30th October 2017. Retrospectively registered.

**Electronic supplementary material:**

The online version of this article (10.1186/s12876-018-0819-6) contains supplementary material, which is available to authorized users.

## Background

Availability of techniques for multi-parallel sequencing [1] and for metabolomic [2] studies has markedly enhanced our ability to study the profile and function of microbiota at various body sites. This has led to identification of alterations in gut microbiota, often referred to as ‘dysbiosis’, in several diseases, including metabolic disorders (e.g. diabetes mellitus, obesity) [3, 4] immunological diseases (inflammatory bowel disease, rheumatological disease, allergic disorders) [5, 6], neurological illnesses (autism, multiple sclerosis) [7] and gastrointestinal and liver diseases (celiac disease, liver cirrhosis) [8, 9]. The role of altered gut microbiota in these various disease states is believed to be mediated, at least partly, by their influence on host’s innate and adaptive immune responses [10, 11].

Attempts have been made to modulate gut microbiota by administration of probiotics or prebiotics, or fecal transplantation [12]. Of these, probiotics, defined as ‘live microorganisms which when administered in adequate amounts confer a health benefit on the host’, appear to be particularly attractive [13]. Short courses of probiotics are widely used in patients with acute gastroenteritis, antibiotic-associated diarrhea, *Helicobacter pylori* infection, etc. [12]. In addition, these preparations are also used for treatment of several immune-inflammatory conditions, such as inflammatory bowel disease, juvenile arthritis and rheumatoid arthritis [14–16]. In animal models, amelioration of inflammation following administration of gut commensals is associated with a change in the composition of the gut microbiota, and in the host immune responses [17]. However, information on whether probiotic administration indeed leads to a change in the composition of gut microbiota and in host immune response is quite limited.

The primary objective of the study was to assess the effect of administration of a probiotic for 4 weeks in healthy women, on the profile of gut microbiota and immune response. A secondary objective was to assess the persistence of these changes, if any, after probiotic administration had been discontinued.

## Methods

### Subjects

The present study had an open-label, single-arm study design, and was conducted in the Department of Gastroenterology of a tertiary-level health care institution in India. Our study adheres to CONSORT guidelines. We enrolled healthy, non-pregnant women as volunteers. Those women who had (i) a systemic (diabetes, autoimmune disease, cancer), gastrointestinal or liver disease that is known to be associated with alteration in intestinal microbiota, (ii) obesity or malnutrition (body mass index of < 18.5 or > 25 Kg/m^2^), (iii) history of taking an anti-microbial agent, probiotic preparation, or a drug that suppresses gastric acid or alters gastrointestinal motility, in the previous 6 weeks, (iv) any inter-current illness in the last 8 weeks, or (v) a recent change in dietary or bowel habits, were excluded.

Each subject provided morning stool and venous blood (6 ml; in lithium heparin tubes) specimens at three time-points, i.e. at baseline (before probiotic administration), after probiotic administration (VSL#3®, one capsule twice a day) for 4 weeks, and at 4 weeks after stopping the probiotic intake. Each capsule contained approximately 112.5 billion live freeze-dried bacteria (a mixture of eight species -- *Streptococcus thermophilus*, *Bifidobacterium breve*, *Bifidobacterium longum*, *Bifidobacterium infantis*, *Lactobacillus acidophilus*, *Lactobacillus plantarum*, *Lactobacillus paracasei*, and *Lactobacillus delbrueckii*), which had been stored at 2–4 °C till ingestion. Stool specimens were frozen immediately after collection and stored at -80 till analysis. Blood was collected for measurement of frequencies of Th1, Th2, Th17 and T regulatory (Treg) cells, for whole blood cultures, and for separation of plasma for measurement of cytokine levels.

Adverse effects, if any, during probiotic administration were recorded. Participants were excluded from analysis, if they did not take the probiotic for the intended duration, or if they received another medication for any reason during the 8-week study period. The study protocol was approved by our institution’s Ethics Committee and written informed consent was obtained from each subject.

### Assessment of composition of gut microbiota

From each stool specimen, bacterial DNA was extracted and V3 region of the 16S rRNA gene was amplified; the resultant DNA library was then subjected to Illumina paired-end sequencing [18]. All specimens were processed in one batch to reduce variability.

The raw paired-end reads, in opposing directions, were trimmed to remove the primer sequences and merged using PANDAseq software [19]; during this step, any sequences that had an overlap in the opposing reads of fewer than 20 nucleotides, provided a merged sequence of shorter than 100 nucleotides, or contained any ambiguous nucleotide were removed. Further, any merged reads with Phred score of < 30 (assessed using NGSQC Toolkit) or with chimeric sequences (identified using Usearch61) were also purged. The remaining high-quality, non-chimeric merged reads were assigned to operational taxonomic units (OTUs) using the UCLUST-based, sub-sampled, open-reference OTU picking protocol of QIIME 1.9 [20]. A representative sequence for each OTU was then aligned with the SILVA core set alignment using the PyNAST tool and a phylogenetic tree was constructed using the FastTree tool [21]. Taxonomy was assigned to each OTU using the QIIME’s UCLUST Consensus Taxonomy Assigner against the SILVA v123 reference OTUs pre-clustered at 97% threshold, using the software’s default parameters [20]. During this step, sequences that failed to align, singleton OTUs (those with only one read in all the specimens taken together), unassigned OTUs, and eukaryotic (chloroplast and mitochondrial) OTUs were removed. Further, to reduce data noise, any OTUs observed in fewer than 10% of stool specimens were purged. Specimen-wise observation counts of various OTUs were tabulated in an OTU table in the ‘biom’ format (referred to hereafter as ‘filtered OTU table’).

#### Beta-diversity analysis

The filtered OTU table was converted into a classic table format, where each row represented an OTU and each column represented a fecal specimen. The cells contained observation counts for a particular OTU in a particular specimen, which were normalized using a log-frequency transformation, as follows:

Normalized value = Log_10_$\left(\frac{OC}{n}\times \frac{\sum X}{N}+1\right)$, where ‘OC’ represented the actual observed count of reads for a particular OTU in a specimen, ‘n’ was the sum of observed read counts for all OTUs in that specimen (column total), Σx was the sum of ‘n’ across all specimens (sum of column totals) and N was the number of specimens.

Beta diversity was then assessed using principal co-ordinate analysis (PCoA) based on weighted UniFrac distance matrices.

#### Alpha-diversity analysis

Since species richness is affected by the depth of sequencing, the OTU table for each specimen was rarefied to identical depth, i.e. the number of reads in the specimen with the fewest reads, using PhyloSeq [22] (v1.12.2). Measures of alpha diversity (number of observed species, and Chao 1 and ACE indices which measure species richness, and Shannon and Simpson indices which represent richness and evenness of taxa) [23] were estimated using PhyloSeq, and compared between time-points using compare_alpha_diversity.py script of QIIME 1.9, using a non-parametric test with Bonferroni correction for multiple comparisons.

#### Analysis of paired data (baseline versus week 4; week 4 versus week 8)

For comparison of data before and during probiotic administration, abundances of individual taxa were compared using Wilcoxon’s matched-pairs signed-rank test with false discovery rate (FDR) correction. Taxa with corrected *P* values ≤0.05 were considered as showing a significant change. Similarly, data during probiotic administration and at 4 weeks after stopping probiotics were also compared.

### Assessment of immune responses

#### Enumeration of Th1, Th2, Th17 and Treg frequencies in blood

Th1, Th2 and Th17 frequencies were determined using intracellular staining followed by flow cytometry [24]. Whole blood (500 μl) was diluted 1:1 with RPMI medium supplemented with 10% fetal bovine serum and 1% antibiotic, and cultured with 50 ng PMA (Sigma, St Louis, MO, USA), 1 μg/ml ionomycin (Sigma, USA) and 10 μg/ml brefeldin-A (Sigma, USA) at 37°C in 5% CO_2_ for 6 h. Then, the cells were first surface-stained with anti-CD4-FITC and anti-CD3-APC antibodies, followed by fixation and permeabilization using cytofix/cytoperm kit (BD Biosciences, Franklin Lakes, NJ, USA). The permeabilized cells were then treated with anti-IFNγ-PE, anti-IL-17A-PerCP-Cy5.5 or anti-IL-4-PE intracellular antibodies. The Th1, Th2 and Th17 cells were then counted as CD4^+^/IFNγ^+^ cells CD4^+^/IL-4^+^ cells and CD4^+^/IL-17A^+^ cells, respectively, in the CD3 gate.

For Treg enumeration, Foxp3 staining kit (BD Pharmingen, USA) was used. In brief, dual surface staining for anti-CD4-FITC and anti-CD25-APC, and intracellular staining for anti-Foxp3-PE was done as per the manufacturer’s instruction. The data were acquired using a BD Canto II (BD Biosciences) flow cytometer and FACS Diva software. CD25^+^/Foxp3^+^ cells in CD4^+^ gate were considered as Tregs [24]. An unstained tube was used as negative control for each specimen.

#### Measurement of plasma cytokine levels

Plasma levels of various cytokines (IFN-γ, IL-12p70, IL-4, IL-10, IL-6 and TNF) were measured using sandwich enzyme-linked immunosorbent assays (ELISA) (Duo-Set ELISA kit; BD Biosciences, San Diego, CA, USA) as per the manufacturer’s instructions. The detection ranges of assays for IL-12p70, IL-10, IL-4 and TNF were 7.8 to 500 pg/ml, and those for the IFN-γ and IL-6 assays were 4.7 to 300 pg/ml. For all the cytokines, absorbance was read at 450 nm using an ELISA plate-reader (Bio-Rad Laboratories, Hercules, CA, USA), and cytokine concentrations were calculated using Bio-Rad microplate manager software. Any values below the detection limit were treated as zero during further analysis.

#### Measurement of cytokine production by immune cells

Heparinized blood was diluted 1:10 in complete RPMI and 1-ml cultures were set up in the presence of plate-bound anti-CD3 (1 μg/ml, catalogue number 16–00-3785, eBiosciences, San Diego, CA, USA) and anti-CD28 (1 μg/ml, catalogue number 16–02-8885, eBiosciences) for stimulation of T cells. Similarly, 1-ml cultures for stimulation of monocytes were set up in separate wells, by adding lipopolysaccharide (LPS: 2 μg/ml, Sigma) [25]. Culture supernatants were harvested at 72 h and stored at − 80°C, till analysis.

Concentrations of various cytokines in the culture supernatant were measured using a human Th1/Th2/Th17 Cytometric Bead Array kit (BD Biosciences) as per the manufacturer’s instructions. The data were acquired using BD FACSCanto II and were analyzed using FCAP (Flow Cytometric Analysis Program) Array v3 software (Soft Flow Hungary Ltd., Pecs, Hungary).

#### Statistics

We had planned to include 20 women in this study. This sample size was based on the usual number of subjects included in such exploratory studies. Immune response data from baseline, first follow-up and second follow-up specimens were expressed as medians and ranges. As a primary analysis, to determine whether VSL#3 intake induced any change in microbiota or immune responses, we compared the data before and after VSL#3 administration (i.e. baseline and first follow-up, respectively) using Wilcoxon signed-rank test, a non-parametric test for paired data. Our secondary objective was to determine whether the changes due to VSL#3 were sustained or not. For this, data from the first and the second follow-up were compared in a similar manner.

## Results

### Study subjects

**Fig. 1:**
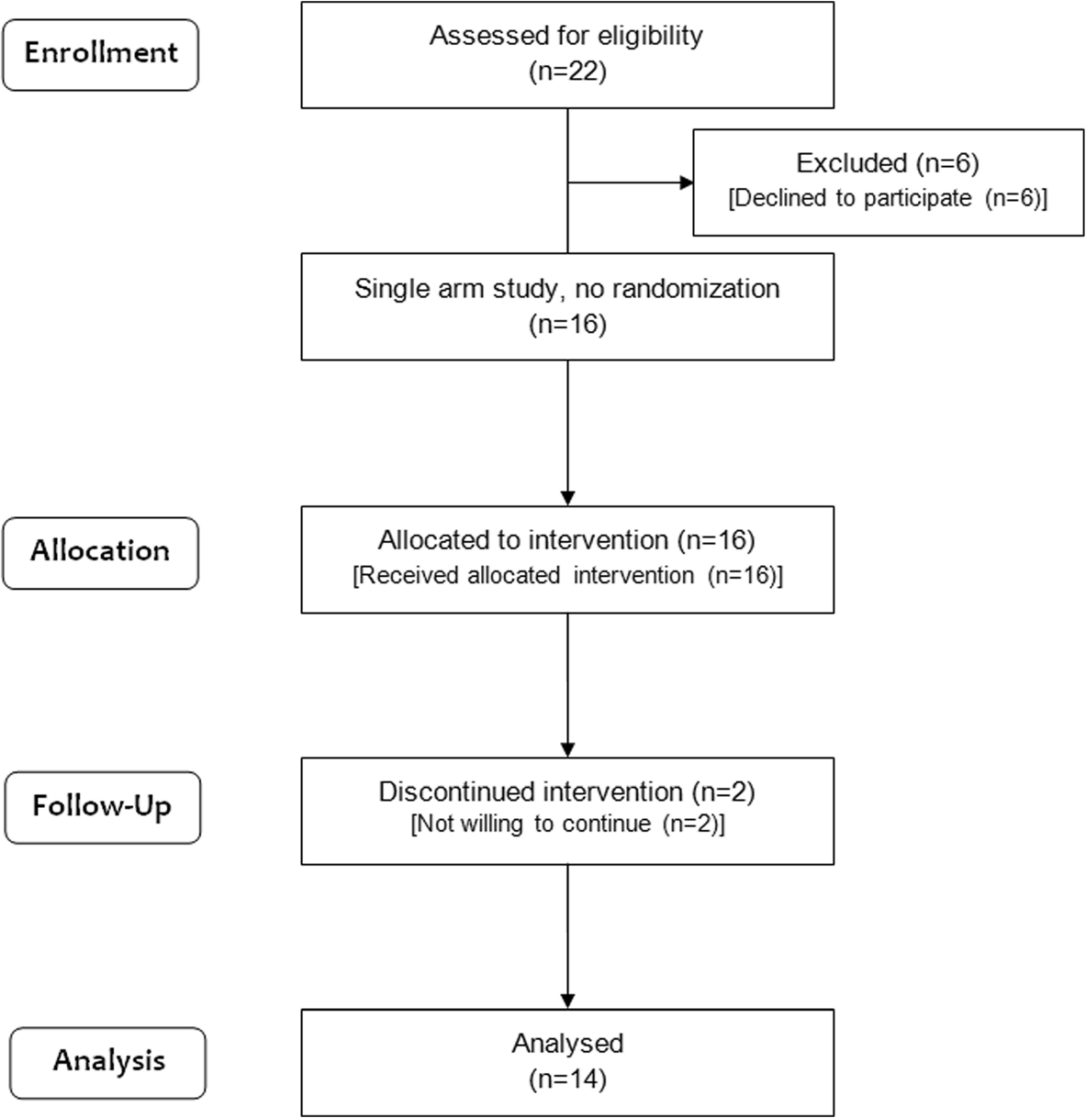
CONSORT flow diagram

The enrolment to the study began in October 2014, and by the end of December 2015, we could enrol only 16 women against the planned sample size of 20. Two of them withdrew from the study before completing the intended duration of probiotic intake. Thus, the data from 14 women, median age (25.5 [18-40] years), were included in the final analysis (Fig. 1). They enroled and complied with 4 weeks of probiotic administration, and provided stool and blood specimens at baseline and after probiotic administration; however, follow-up stool specimen after discontinuation of probiotic was not available for one subject. Hence, a total of 41 stool specimens (14 each at baseline and week 4, and 13 at week 8) and 42 blood specimens (14 each at three time-points) were available for analysis. All the women consumed predominantly vegetarian diet before, during and after probiotic use.

### Composition of gut microbiota

Median [range] numbers of high-quality sequence reads were similar for stool specimens collected at the three time-points, i.e. 266,886 (*n* = 14; range 235,668–311,683; total reads 582,236); 264,915 (n = 14; 151,651–354,997; total reads 3,729,837) and 243,739 (*n* = 13; 193,539–330,165; total reads 3,295,921), respectively. These reads belonged to 12,550 non-singleton OTUs; of these, data for 1115 OTUs which were identified in at least four specimens each were analyzed further.

The five most abundant bacterial phyla in the baseline stool specimens were Firmicutes (45.10% [20.05–62.34%]), Bacteroidetes (39.11% [3.58–76.23%]), Proteobacteria (6.73% [0.12–29.23%]), Actinobacteria (2.08% [0.59–19.84%]) and Tenericutes (0.13% [0.00–7.05%]).

On PCoA, no separation was noticed between the stool specimens collected at the three time-points, i.e. baseline, during probiotic intake and after stopping the probiotic (Additional file 1: Figure S1A and B). Furthermore, neither the comparison of specimens collected before and after probiotic administration (Additional file 2: Figure S2A), nor that of specimens collected during and after stopping probiotic administration (Additional file 2: Figure S2B) showed any change in alpha diversity.

**Fig. 2:**
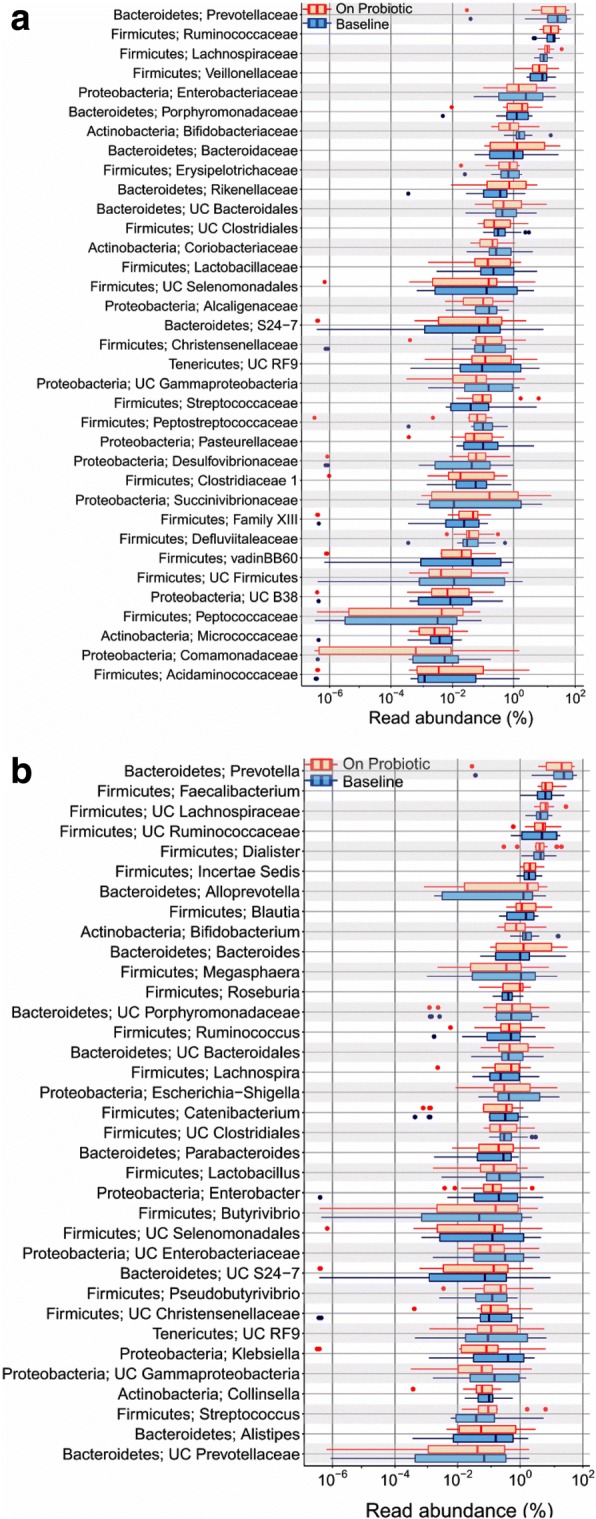
Abundances of various bacterial families (**a**) and genera (**b**) in fecal microbiota from healthy women before (blue) and after (red) four weeks of probiotic administration. Data are shown using box-plots and percent values on a log_10_ scale. The ends of boxes represent 25th to 75^th^ centiles, and any dots to the left or right of the boxes indicate outliers

**Fig. 3:**
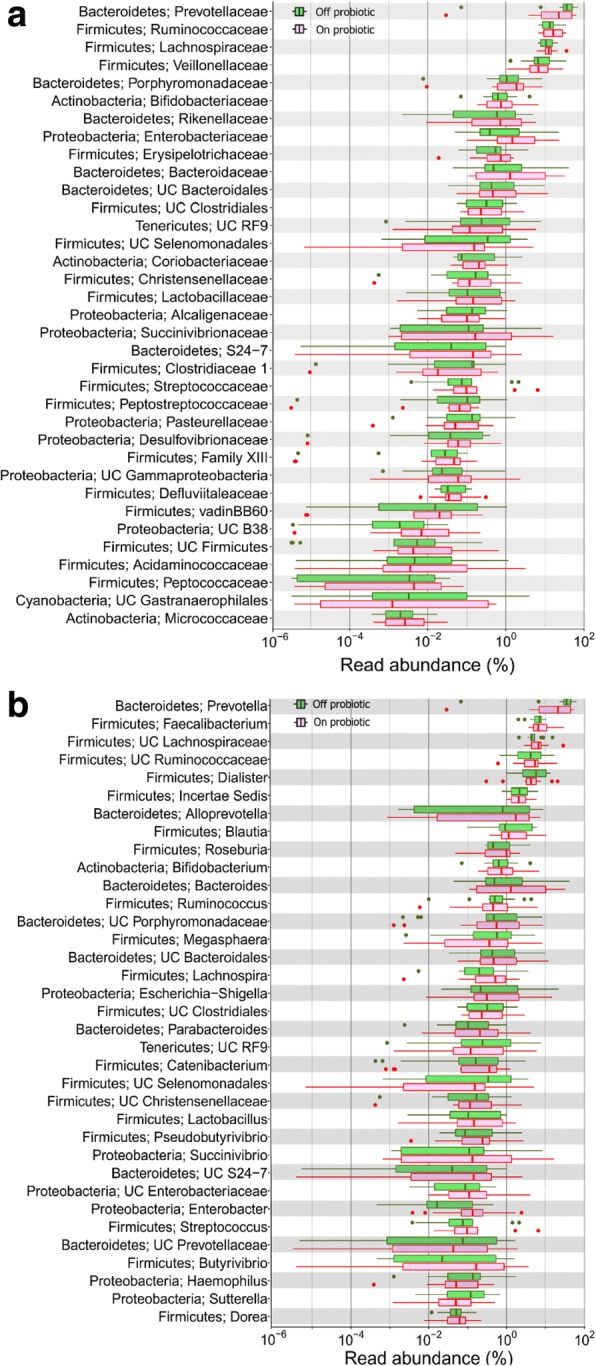
Abundances of bacterial families (**a**) and genera (**b**) in fecal microbiota from healthy women after four weeks of probiotic administration (red) and 4-weeks after discontinuation of probiotic administration (green). Data are shown using box-plots and percent values on a log_10_ scale. The ends of boxes represent 25th to 75^th^ centiles, and any dots to the left or right of the boxes indicate outliers

Abundances of various bacterial taxa in the stools collected at baseline and during probiotic administration showed no differences at phylum, class, order, family and genus levels (Fig. 2, Additional file 3: Table S1). Similarly, a comparison of abundances of various bacterial taxa in the specimens obtained during probiotic administration and 4 weeks after its discontinuation showed no difference (Fig. 3, Additional file 3: Table S1).

### Immune responses

#### Enumeration of T cell subsets

**Fig. 4:**
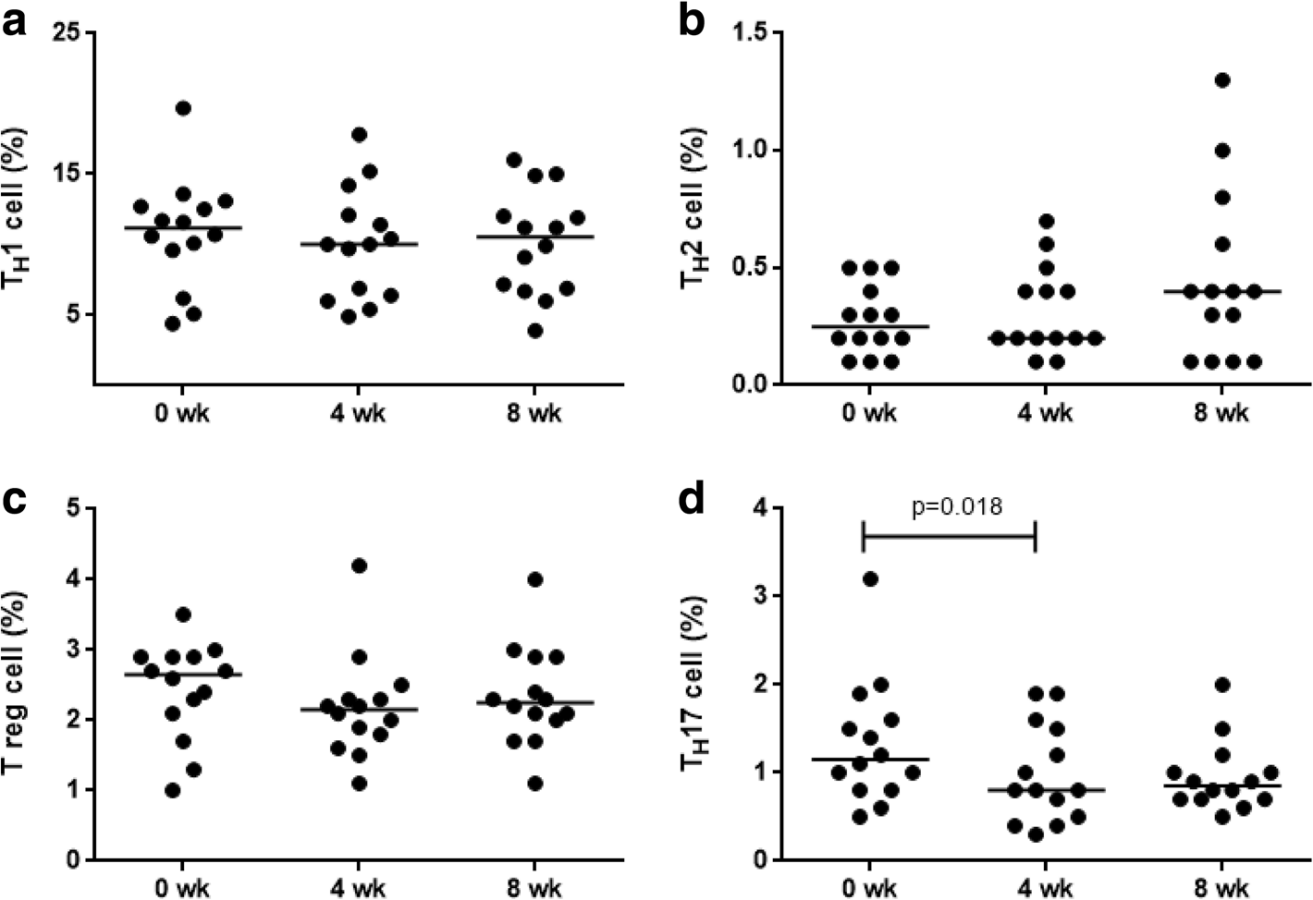
Frequencies of Th1 (**a**), Th2 (**b**), Treg (**c**) and Th17 cells (**d**) in whole blood cultures from healthy women (*n* = 14) at baseline, after 4-weeks of probiotic administration and 4-weeks after discontinuation of probiotic administration (8 wk) by flow cytometry (Mann-Whitney U test)

The effect of probiotic administration on various T-cell subsets, namely Th1, Th2, Th17 and Treg cells, was assessed by enumerating these cells at baseline, after 4 weeks of probiotic administration and 4 weeks after stopping probiotic administration (Additional file 4: Figure S3 and Additional file 5: Figure S4). There was no difference in the proportion of Th1 (Fig. 4a), Th2 (Fig. 4b) and Treg cells (Fig. 4c) between the three time-points. The proportion of Th17 cells (1.15% ± 0.67% vs 0.80% ± 0.53%; *p* = 0.018) (Fig. 4d) showed a significant reduction after probiotic administration.

#### Cytokine levels in plasma and culture supernatants

Comparison of plasma levels of IFN-γ, IL-4, IL-6, IL-10 and TNF before and after 4 weeks of probiotic administration did not reveal any difference. The cytokine levels for most of the subjects were below detection limit.

Supernatants from whole blood cultures stimulated with anti-CD3 and anti-CD28 did not show any significant change in the amount of IFN-γ, IL-4, IL-6, IL-17, IL-2 or TNF after probiotic administration (Table 1).

**Table 1:** Cytokine levels in supernatants of whole-blood CD3^+^- and CD28^+^-stimulated cultures from healthy women (*n* = 14)

Cytokine	Before probiotic administration	After probiotic administration for four weeks	Four weeks after stopping probiotic administration
IL-2	23.4 (1.7–210.2)	38.7 (0–673.3)	17.4 (0.9–44.3)
IL-4	3.9 (0–16.0)	4.3 (0–35.4)	2.8 (0–19.0)
IL-6	4009 (393–12,954)	4835 (163–10,068)	10,791 (2073–14,754)
IL-10	41.9 (4.7–1664.0)	37.3 (5.5–410.3)	99.2 (17.6–481.6)
TNF	125.2 (23.5–584.8)	103.4 (15.9–487.9)	258.3 (68.8–1191.2)
IFN-γ	30.8 (8.3–309.8)	23.5 (0–416.5)	34.6 (0–858.8)
IL-17	9.3 (0–47.3)	10.5 (0–57.1)	16.6 (0–58.9)

The levels were measured using cytometric bead array. All data are shownin pg/ml and as median (range)

**Fig. 5:**
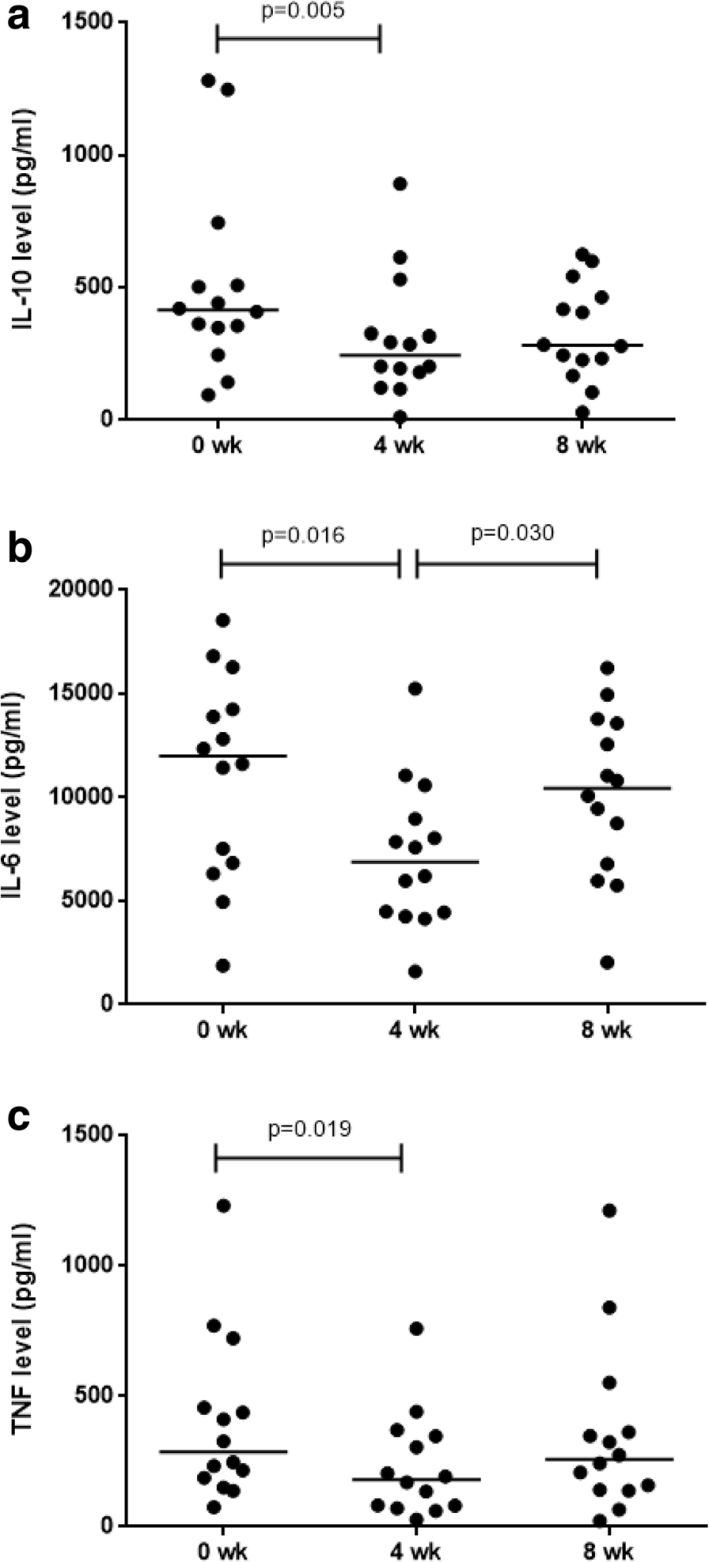
Estimation of levels of cytokines (pg/ml): IL-10 (**a**), IL-6 (**b**) and TNF (**c**), predominantly produced by monocytes on LPS stimulation of whole blood in culture supernatant of healthy women (*n* = 14). The estimation was done at three time-points; baseline, 4-weeks of probiotic administration and 4-weeks after discontinuation of probiotics by BD Cytometric Bead Array (Mann-Whitney U test)

The median (range) cytokine levels in supernatants from LPS-stimulated whole-blood cultures showed a significant reduction in IL-10 (414.0 [94.7–1279.8] pg/ml versus 244.0 [12.1–891.4] pg/ml; *p* = 0.005), IL-6 (11,978 [1882–18,541] pg/ml vs 6895 [1590–15,250] pg/ml; *p* = 0.016) and TNF (287.0 [74.6–1229.4] pg/ml vs 181.0 [26.9–758.4] pg/ml; *p* = 0.019) levels. IL-6 levels returned to baseline after probiotic discontinuation (10,448 [2040–16,252] pg/ml) (Fig. 5). However, there was no difference in the levels of IL-2, IL-4, IFN-γ and IL-17 cytokines at the three time points (Table 2).

**Table 2:** Cytokine levels in supernatants of whole-blood lipopolysaccharide-stimulated cultures from healthy women (*n* = 14)

Cytokine	Baseline		4-weeks		8-weeks	
IL-2	0.61	(0–2.71)	0.57	(0–2.10)	0.41	(0–7.30)
IL-4	0.86	(0–15.00)	1.16	(0–2.16)	0.08	(0–3.06)
IFN-γ	1.03	(0–4.67)	0.44	(0–3.94)	0.13	(0–5.33)
IL-17	15.65	(0–46.12)	10.17	(0–48.46)	14.21	(0–81.49)

The levels were measured using cytometric bead array. All data are shownin pg/mland as median (range)

## Discussion

We studied the effect of administration of a probiotic preparation on composition of stool microbiota, as also on various measures of immune responses in a cohort of healthy women. Contrary to our expectations, probiotic administration failed to induce any change in the diversity and composition of gut microbiota. Furthermore, the changes in immune responses were limited to a reduction in in vitro production of cytokines which are produced predominantly by monocytes in response to stimulation with LPS, and a decrease in the frequency of Th17 cells, with no change in the frequency of Th1, Th2 or Treg cells, plasma cytokine levels, or cytokine production in response to stimulation of T cells.

The microbiota is believed to play an intimate role in regulation and maintenance of normal intestinal physiology, metabolism and immune functions. The main bacterial phyla in baseline specimens in our subjects, in the decreasing order of abundance, were Firmicutes, Bacteroidetes, Proteobacteria and Actinobacteria. This was similar to the pattern reported from developed countries [26], other Asian countries [27, 28] and India [29].

Probiotics are believed to restore the ‘dysbiotic’ gut microbiota to a state closer to normal [30] and to modulate the host immune responses. VSL#3 is a commonly used probiotic preparation that contains eight bacterial species considered to be beneficial to humans. In some studies, its use in patients with ulcerative colitis [31], pouchitis [32] and irritable bowel syndrome [33] was found to lead to changes in gut microbiota. These changes included an increase in the abundance of lactobacilli and bifidobacteria, species that are present in this preparation [32], and specific detection in stool or tissue biopsies of the bacterial species contained in this preparation [33]. In addition, in one study, there was a decline in the abundance of Bacteriodes [33], and another study showed an increase in bacterial diversity [32]. However, in our study, administration of VSL#3 did not lead to any change in either the composition, i.e. relative abundance of various taxa, or in the diversity of gut microbiota. There could be several explanations for this lack of change. First, the gut microbial community is known to be quite stable, and is rapidly restored on removal of an external influence, such as acute inflammation or antibiotic administration. It is possible that the gut microbiota in our volunteers changed on administration of VSL#3, but that this change was transient and was reversed despite its continued administration for 4 weeks, when the follow-up specimens were collected. Second, our study included healthy volunteers with normal microbiota and not patients. It is possible that probiotics can induce change in the abnormal gut microbiota of diseased persons, but not in normal microbiota of healthy persons.

In a previous study, VSL#3 administration to healthy persons led to an increase in Lactobacillus, Bifidobacterium, and Bacteroides species; however, the dose used in that study was four-fold higher than that used in our study [33]. In another study among healthy elderly subjects (aged 65–85 years), VSL#3 supplementation in a dose similar to our study, but for 8 weeks, failed to show any change in the abundance of various Bifidobacteria [34]. Also, in patients with juvenile arthritis, VSL#3 supplementation in a dose similar to ours for 3 months did not lead to any significant change in gut microbiota [35]. Whether these differences between various studies are related to the dose of probiotic used remains unclear.

Administration of VSL#3 in our study led to a decrease in the production of various cytokines (IL-6, TNF, IL-10) in ex-vivo LPS-stimulated whole-blood cultures. In a recent study, ingestion for 4 weeks of a probiotic that contained *Lactobacillus casei* led to a reduction in the frequency of IL-6-producing monocytes on ex-vivo LPS stimulation [36]. However, in some older studies, supplementation with lactobacilli in healthy adults had no effect on IL-6 production on LPS stimulation [37, 38]. Administration of *Lactobacillus rhamnosus* GG or of *Bifidobacterium animalis* subspecies lactis to healthy adults has also been shown to reduce TNF-α production in whole-blood cultures [37–39]. In in vitro co-culture experiments, VSL#3 led to an increase in the frequency of colonic and blood dendritic cells that produced IL-10 [40]. Bifidobacteria are also known to induce IL-10 production [41]. However, in contrast, we found a significant reduction in LPS-induced IL-10 production in whole-blood cultures after VSL#3 administration. This may be because of an opposing effect of *Lactobacillus casei*, another constituent of VSL#3, intake of which has previously been shown to reduce IL-10 production in whole-blood cultures from healthy adults [42].

Lack of effect of probiotic administration on plasma cytokine levels in our study is similar to that observed in several previous studies [43, 44]. Moreover, the cytokine responses may vary depending on the probiotic preparation used [45]. With preparations such as VSL#3, which contain a mixture of organisms, the stimulatory effect of some organisms may be cancelled out by the inhibitory effect of the other constituents [38]. Thus, future studies on effect of probiotics on immune responses may need to focus on individual probiotics, rather than their mixtures. Further, it is possible that the effect of probiotics on immune responses in healthy persons differs from that in various disease states. Such a difference could explain why we failed to find a change in immune responses following VSL#3, though some previous clinical studies have shown this preparation to be useful in some inflammatory conditions.

Probiotic administration did not lead to a change in the frequencies of Th1, Th2 or Treg cells in our subjects. In mouse, VSL#3 preparation has been shown to shift a Th2-polarized response to a Th1/Treg type response [46]. It is possible that normal Th1/Th2 homeostasis is not amenable to a change by probiotic administration; by contrast, in disease states such as IBD, where this ratio is disturbed, probiotics may help restore this balance towards normal.

A reduction in Th17 cells observed after probiotic usage, similar to that observed in our study, has also been reported in animal models and in colonic tissue from patients with IBD [47]. Intestine is rich in Th17 cells, which are known to secrete pro-inflammatory cytokines such as IL-6, TNF-α, IL-22 and IL-23 and play a role in the pathogenesis of IBD [47]. An imbalance in gut microbiota may lead to immune activation through an expansion of Th17 cells [48]. For instance, gut dysbiosis may provide an increased amount of TLR ligands, leading to activation of dendritic cells or monocytes to produce IL-6 and TGF-β, which may in turn mediate differentiation of Th17 cells.

We did not observe any change in the frequency of Tregs after VSL#3 administration. By contrast, in patients with IBD, the use of probiotics that contain either one (such as *Lactobacillus casei* BL23 or *Bifidobacterium infantis*) or several (e.g. VSL#3) bacterial species, or of prebiotics (e.g. lactic acid) has been shown to increase the frequency of Treg cells [49, 50]. This difference could be related to the fact that probiotics restore the immune milieu towards normal in disease states, but have no effect when the milieu is healthy.

Our study has some limitations. First, gut microbiota are known to vary across geographical  regions, and with dietary habits, etc. Since we studied subjects in only one population group, it may be difficult to extrapolate our data to other population groups. Thus, further similar studies in other populations may be warranted. Further, our study included only young women. This was done to ensure homogeneity of the study subjects, and it is unlikely that this by itself would have influenced the effect of probiotic administration.

## Conclusions

Overall, we found that administration of VSL#3 did not lead to any change in gut microbiota or in the frequencies of Th1, Th2 and Treg cells in healthy women. Hence, it is unlikely that this probiotic preparation can be used to induce changes in the composition of gut microbiota in healthy subjects. However, it led to a reduction in the frequency of Th17 cells and in the production of pro-inflammatory cytokine from monocytes. These immune changes may be involved in its beneficial effect in immunoinflammatory disorders.

## Additional files

Additional file 1: **Figure S1.** Beta diversity using principal co-ordinate analysis of weighted UniFrac distances of specimens collected from healthy women. **a** At baseline (blue) and after four weeks of probiotic administration (red). **b** After four weeks of probiotic administration (red) and 4 weeks after stopping probiotic administration (green). (TIF 1555 kb)

Additional file 2: **Figure S2.** Comparison of measures of alpha diversity in specimens from healthy women. **a** Comparison of specimens collected at baseline (blue) and after four weeks of probiotic administration (red). **b** Comparison of specimens collected after four weeks of probiotic administration (red) and 4 weeks after stopping probiotic administration (green). (TIF 1925 kb)

Additional file 3: **Table S1.** Comparison of abundance of various bacterial groups at phylum, class, order, family and genus levels in stool specimens collected before probiotic administration (week 0), during probiotic administration (week 4) and 4 weeks after stopping probiotic administration (week 8). (DOCX 101 kb)

Additional file 4: **Figure S3.** Representative flow cytometry plots of IFN-γ^+^Th1, IL-4^+^ Th2 and IL-17^+^ Th17 cells in the peripheral blood of healthy subjects. Percentages of CD4^+^/ IFN-γ^+^/ Th1, CD4^+^/ IL-4^+^/Th2 and CD4^+^/ IL-17^+^/ Th17 cells were determined in CD3 gate. (TIF 3451 kb)

Additional file 5: **Figure S4.** Representative flow cytometry plot of T regulatory  (Treg) cells in the peripheral blood of healthy subjects. CD25^+^/FOXP3^+^ cell percentages were determined in CD4 gate. (TIF 2938 kb)

## Abbreviations

ACE: Abundance-based coverage estimate
APC: Allophycocyanin
FITC: Fluorescein isothiocyanate
IBD: Inflammatory bowel disease
LPS: Lipopolysaccharide
OTU: Operational taxonomic unit
PCoA: Principal co-ordinate analysis
PE: Phycoerythrin
PMA: Phorbol myristate acetate
RPMI: Roswell Park Memorial Institute medium
Treg: T regulatory cell
