# Supplementary figures and images for: Effect of administration of a probiotic preparation on gut microbiota and immune response in healthy women in India: an open-label, single-arm pilot study
Source: BMC Gastroenterol. 2018 Jun 15;18:85. doi: 10.1186/s12876-018-0819-6 (PMC6003164; doi:10.1186/s12876-018-0819-6)

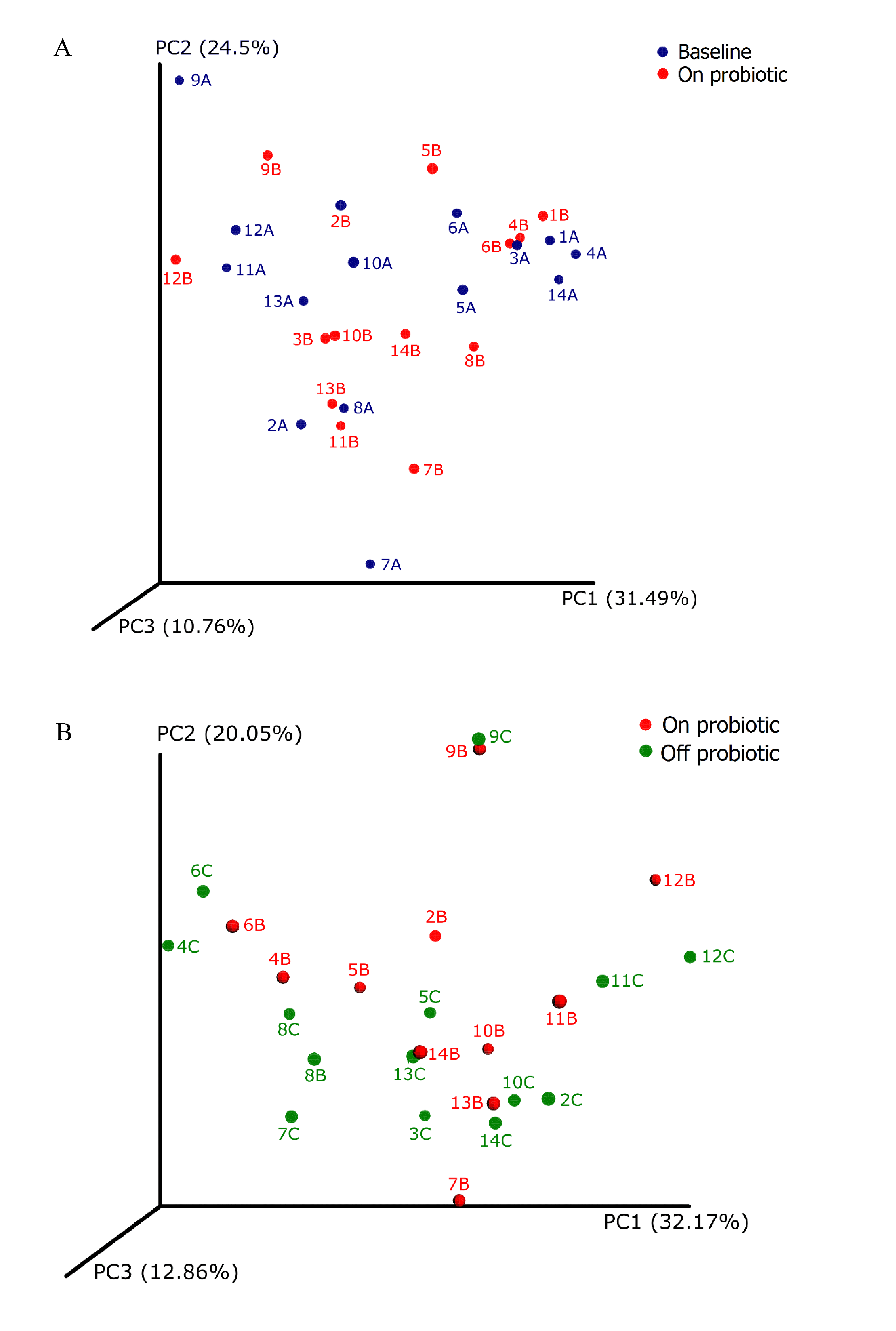

Supplement: Supplementary file 1 — Figure S1. Beta diversity using principal co-ordinate analysis of weighted UniFrac distances of specimens collected from healthy women. a At baseline (blue) and after four weeks of probiotic administration (red). b After four weeks of probiotic administration (red) and 4 weeks after stopping probiotic administration (green). (TIF 1555 kb) [file 12876_2018_819_MOESM1_ESM.tif]

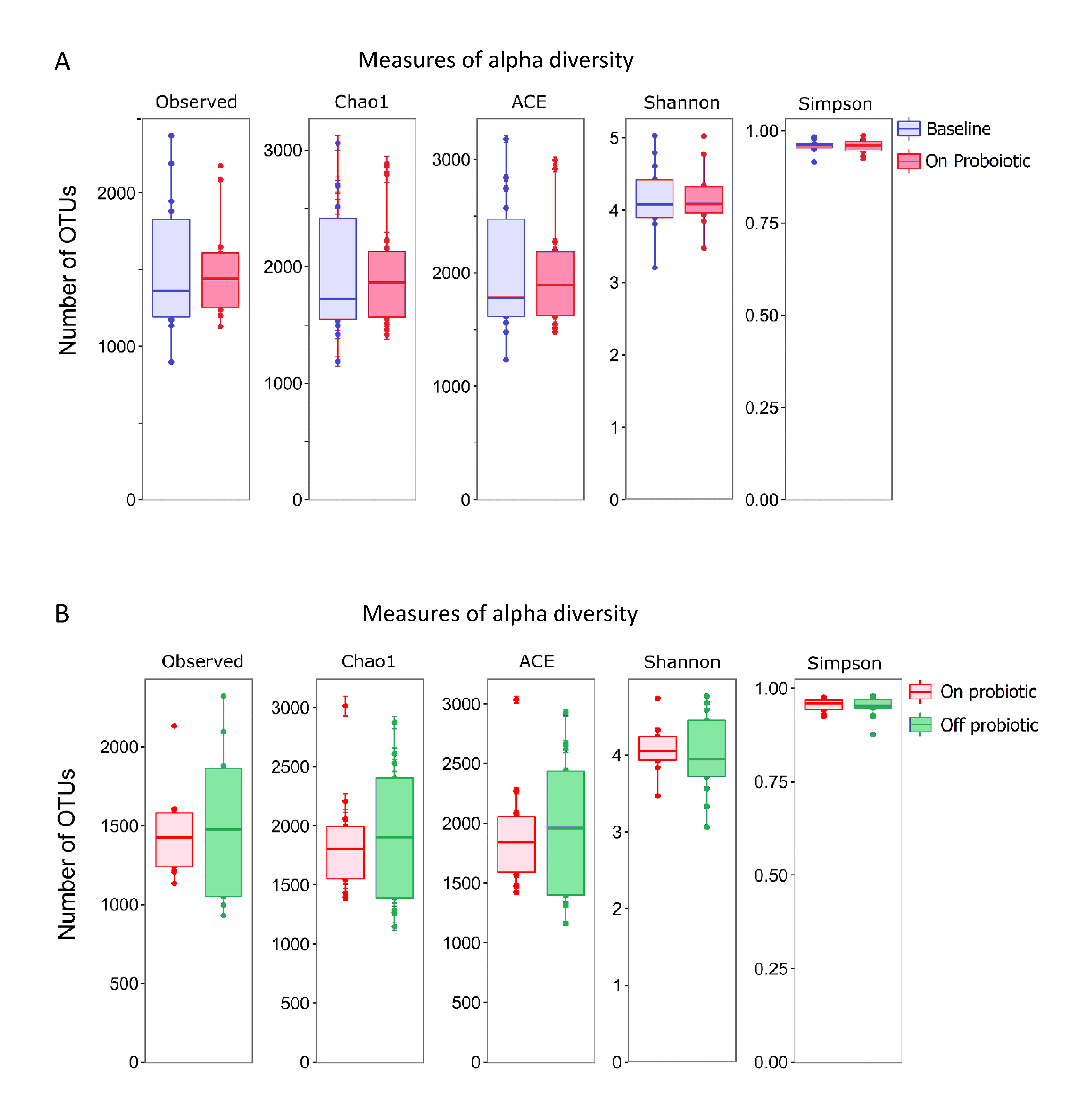

Supplement: Supplementary file 2 — Figure S2. Comparison of measures of alpha diversity in specimens from healthy women. a Comparison of specimens collected at baseline (blue) and after four weeks of probiotic administration (red). b Comparison of specimens collected after four weeks of probiotic administration (red) and 4 weeks after stopping probiotic administration (green). (TIF 1925 kb) [file 12876_2018_819_MOESM2_ESM.tif]

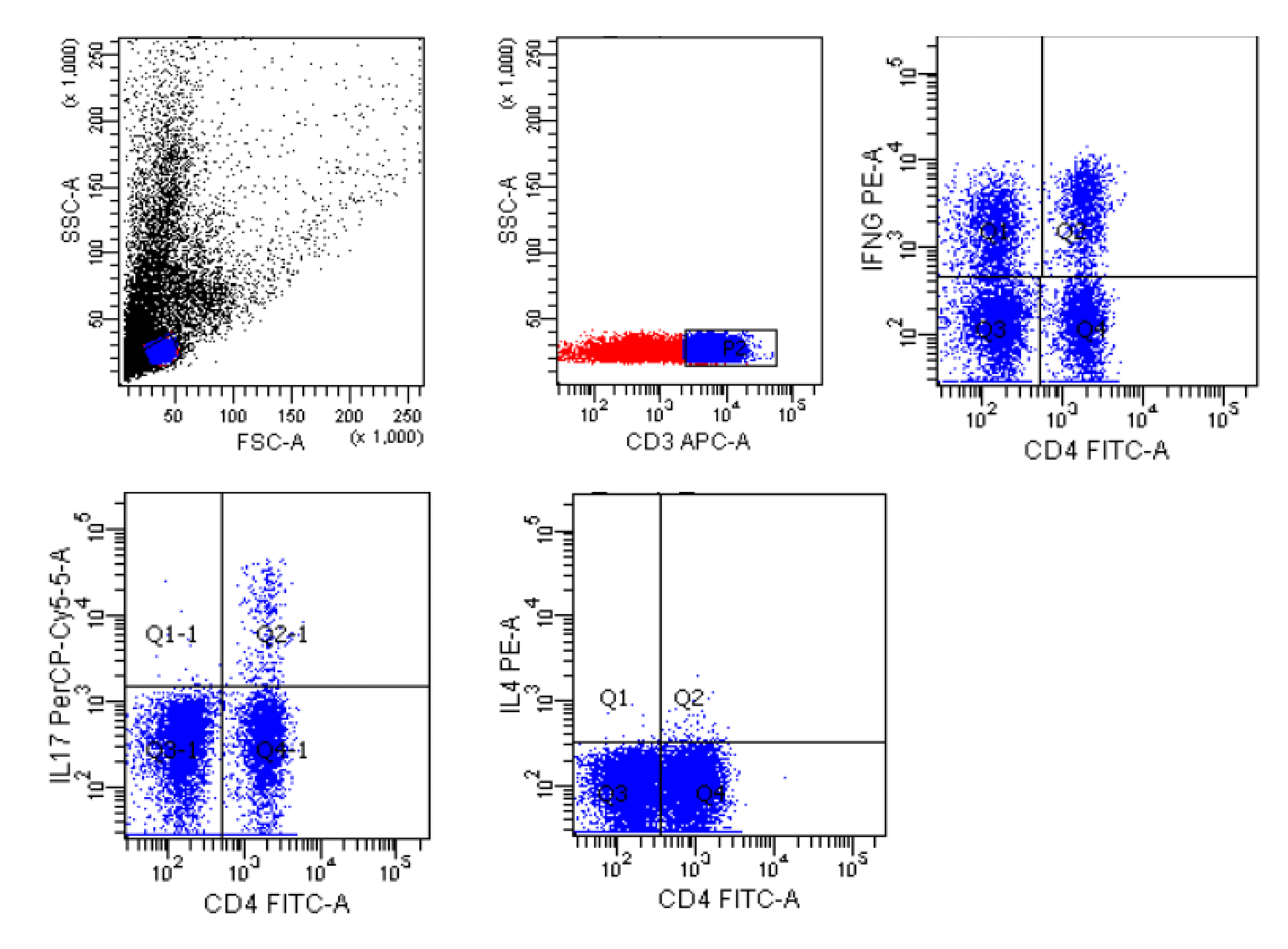

Supplement: Supplementary file 4 — Figure S3. Representative flow cytometry plots of IFN-γ+Th1, IL-4+ Th2 and IL-17+ Th17 cells in the peripheral blood of healthy subjects. Percentages of CD4+/ IFN-γ+/ Th1, CD4+/ IL-4+/Th2 and CD4+/ IL-17+/ Th17 cells were determined in CD3 gate. (TIF 3451 kb) [file 12876_2018_819_MOESM4_ESM.tif]

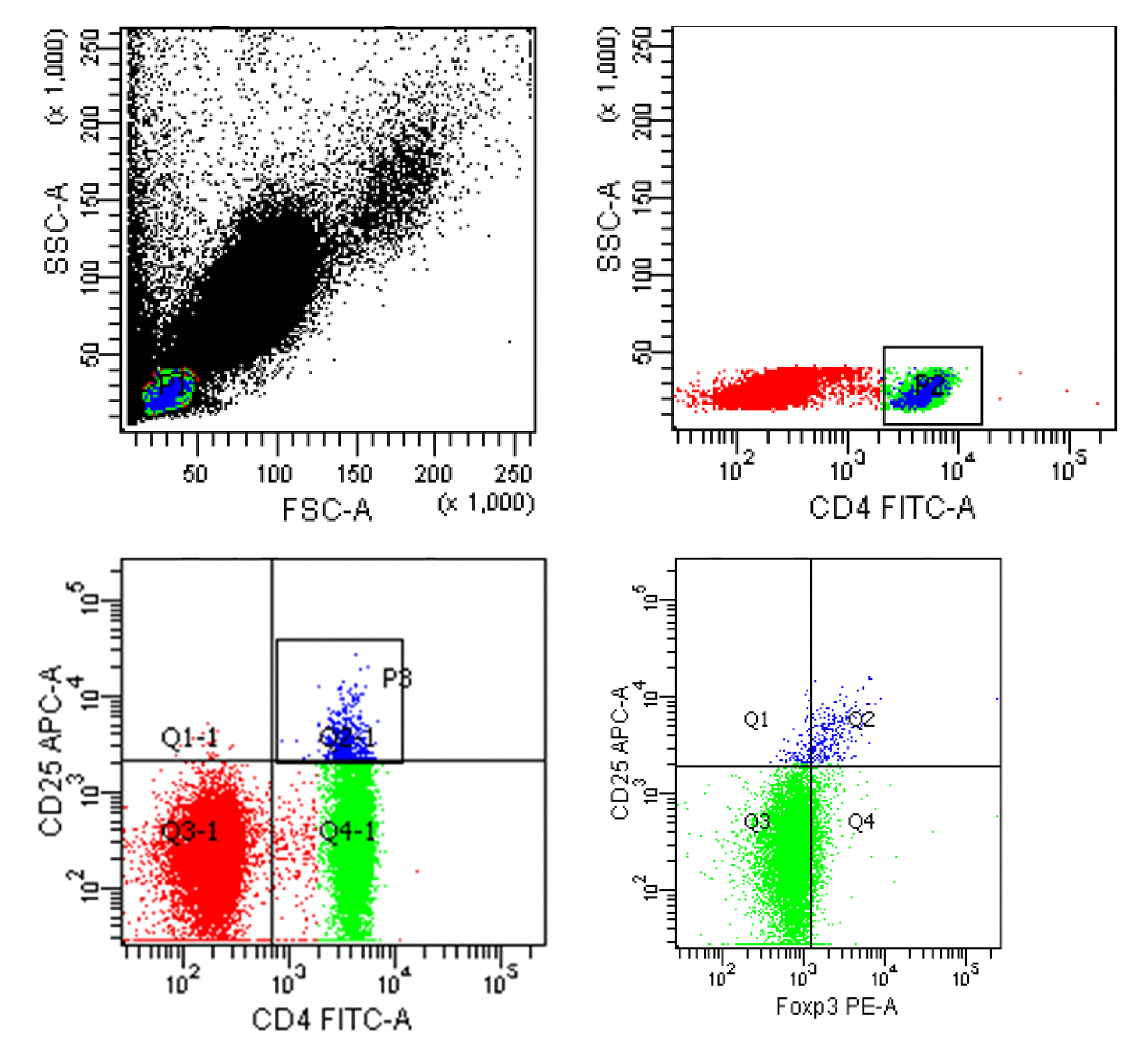

Supplement: Supplementary file 5 — Figure S4. Representative flow cytometry plot of T regulatory (Treg) cells in the peripheral blood of healthy subjects. CD25+/FOXP3+ cell percentages were determined in CD4 gate. (TIF 2938 kb) [file 12876_2018_819_MOESM5_ESM.tif]
