# Supplementary material for: Effect of administration of a probiotic preparation on gut microbiota and immune response in healthy women in India: an open-label, single-arm pilot study
Source: BMC Gastroenterol. 2018 Jun 15;18:85. doi: 10.1186/s12876-018-0819-6 (PMC6003164; doi:10.1186/s12876-018-0819-6)
Supplement: Supplementary file 3 — Table S1. Comparison of abundance of various bacterial groups at phylum, class, order, family and genus levels in stool specimens collected before probiotic administration (week 0), during probiotic administration (week 4) and 4 weeks after stopping probiotic administration (week 8). (DOCX 101 kb) [file 12876_2018_819_MOESM3_ESM.docx]

**Supplementary Table 1: Comparison of abundance of various bacterial groups at phylum, class, order, family and genus levels in stool specimens collected before probiotic administration (week 0; W0), during probiotic administration (week 4; W4) and 4 weeks after stopping probiotic administration (week 8; W8). P values as well as Benjamini-Hochberg false discovery rates (FDR) for comparison of abundances before and during probiotic administration, and during and after discontinuation of probiotic administration are shown.**

| **Taxonomic Group** | **W0 vs W4** | | **W4 vs W8** | | **Week0** | | | **Week4** | | | **Week8** | | |
| --- | --- | --- | --- | --- | --- | --- | --- | --- | --- | --- | --- | --- | --- |
|  | p value | FDR | p value | FDR | Median | Min | Max | Median | Min | Max | Median | Min | Max |
| **Phylum** | | | | | | | | | | | | | |
| p_Firmicutes | 0.63 | 0.98 | 0.41 | 0.85 | 45.10 | 20.05 | 62.34 | 52.02 | 26.81 | 73.68 | 36.81 | 19.53 | 74.27 |
| p_Bacteroidetes | 0.76 | 0.98 | 0.34 | 0.85 | 39.11 | 3.58 | 76.23 | 42.17 | 16.10 | 67.72 | 50.38 | 11.40 | 77.79 |
| p_Proteobacteria | 0.27 | 0.98 | 0.04 | 0.48 | 6.73 | 0.12 | 29.23 | 3.96 | 1.17 | 27.06 | 1.99 | 0.91 | 23.96 |
| p_Actinobacteria | 0.01 | 0.16 | 0.45 | 0.85 | 2.08 | 0.59 | 19.84 | 0.97 | 0.27 | 7.00 | 1.14 | 0.13 | 6.67 |
| p_Tenericutes | 0.86 | 0.98 | 0.50 | 0.85 | 0.13 | 4.4E-04 | 7.05 | 0.12 | 1.2E-03 | 5.98 | 0.24 | 8.4E-04 | 7.93 |
| p_Cyanobacteria | 0.64 | 0.98 | 0.85 | 0.91 | 4.4E-03 | 0.00 | 1.51 | 1.4E-03 | 0.00 | 0.57 | 3.3E-03 | 0.00 | 4.04 |
| p_Verrucomicrobia | 0.15 | 0.89 | 0.32 | 0.85 | 3.6E-03 | 0.00 | 1.38 | 5.3E-04 | 0.00 | 0.73 | 9.7E-04 | 0.00 | 0.33 |
| p_Lentisphaerae | 0.50 | 0.98 | 0.91 | 0.91 | 1.5E-03 | 0.00 | 0.19 | 1.1E-03 | 0.00 | 0.01 | 0.00 | 0.00 | 0.10 |
| p_Spirochaetae | 0.90 | 0.98 | 0.43 | 0.85 | 3.6E-04 | 0.00 | 0.22 | 3.9E-04 | 0.00 | 0.94 | 0.00 | 0.00 | 7.02 |
| p_Elusimicrobia | 0.69 | 0.98 | 0.58 | 0.87 | 0.00 | 0.00 | 0.44 | 1.7E-04 | 0.00 | 0.52 | 0.00 | 0.00 | 0.42 |
| p_Fusobacteria | 1.00 | 1.00 | 0.75 | 0.91 | 0.00 | 0.00 | 2.7E-03 | 0.00 | 0.00 | 2.4E-03 | 0.00 | 0.00 | 0.09 |
| p_TM7 | 0.63 | 0.98 | 0.88 | 0.91 | 0.00 | 0.00 | 0.30 | 0.00 | 0.00 | 0.18 | 0.00 | 0.00 | 0.16 |
| **Class** | | | | | | | | | | | | | |
| p_Bacteroidetes  c_Bacteroidia | 0.76 | 1.00 | 0.34 | 0.91 | 39.11 | 3.58 | 76.23 | 42.17 | 16.10 | 67.72 | 50.38 | 11.40 | 77.79 |
| p_Firmicutes  c_Clostridia | 0.43 | 1.00 | 0.45 | 0.91 | 32.66 | 9.57 | 43.83 | 32.79 | 15.75 | 54.09 | 25.80 | 15.66 | 59.34 |
| p_Firmicutes  c_Negativicutes | 0.86 | 1.00 | 0.79 | 0.91 | 8.89 | 3.33 | 28.32 | 8.18 | 3.44 | 29.86 | 6.80 | 1.33 | 34.83 |
| p_Proteobacteria  c_Gammaproteobacteria | 0.76 | 1.00 | 0.02 | 0.25 | 4.72 | 0.07 | 29.14 | 3.56 | 0.38 | 26.07 | 1.48 | 0.19 | 22.89 |
| p_Actinobacteria  c_Actinobacteria | 0.06 | 0.44 | 0.38 | 0.91 | 1.51 | 0.48 | 16.20 | 0.75 | 0.19 | 6.96 | 0.63 | 0.07 | 4.05 |
| p_Firmicutes  c_Erysipelotrichia | 0.17 | 0.80 | 0.89 | 0.91 | 0.69 | 0.03 | 1.90 | 0.74 | 0.02 | 1.59 | 0.54 | 0.06 | 3.75 |
| p_Firmicutes  c_Bacilli | 1.00 | 1.00 | 0.34 | 0.91 | 0.41 | 0.01 | 6.26 | 0.88 | 0.03 | 6.61 | 0.42 | 0.04 | 2.14 |
| p_Proteobacteria  c_Betaproteobacteria | 0.81 | 1.00 | 0.07 | 0.52 | 0.28 | 0.01 | 0.70 | 0.10 | 0.01 | 2.05 | 0.29 | 0.03 | 1.80 |
| p_Actinobacteria  c_Coriobacteriia | 0.06 | 0.44 | 0.59 | 0.91 | 0.27 | 0.03 | 4.22 | 0.20 | 0.04 | 1.12 | 0.07 | 0.04 | 2.62 |
| p_Tenericutes  c_Mollicutes | 0.86 | 1.00 | 0.50 | 0.91 | 0.13 | 4.4E-04 | 7.05 | 0.12 | 1.2E-03 | 5.98 | 0.24 | 8.4E-04 | 7.93 |
| p_Proteobacteria  c_Deltaproteobacteria | 0.24 | 0.80 | 0.62 | 0.91 | 0.04 | 0.00 | 0.98 | 0.06 | 0.00 | 0.76 | 0.04 | 0.00 | 0.39 |
| p_Firmicutes  c_UC Firmicutes | 0.36 | 1.00 | 0.79 | 0.91 | 0.01 | 0.00 | 1.95 | 4.5E-03 | 4.0E-04 | 0.67 | 0.01 | 0.00 | 0.25 |
| p_Cyanobacteria  c_Melainabacteria | 0.64 | 1.00 | 0.85 | 0.91 | 4.4E-03 | 0.00 | 1.51 | 1.4E-03 | 0.00 | 0.57 | 3.3E-03 | 0.00 | 4.04 |
| p_Lentisphaerae  c_Lentisphaeria | 0.50 | 1.00 | 0.91 | 0.91 | 1.5E-03 | 0.00 | 0.19 | 1.1E-03 | 0.00 | 0.01 | 0.00 | 0.00 | 0.10 |
| p_Verrucomicrobia  c_Verrucomicrobiae | 0.21 | 0.80 | 0.73 | 0.91 | 8.4E-04 | 0.00 | 1.38 | 1.7E-04 | 0.00 | 0.73 | 0.00 | 0.00 | 0.02 |
| p_Proteobacteria  c_UC Proteobacteria | 0.03 | 0.44 | 0.01 | 0.23 | 4.4E-04 | 0.00 | 0.04 | 3.0E-03 | 0.00 | 0.08 | 0.00 | 0.00 | 0.09 |
| p_Spirochaetae  c_Spirochaetes | 0.90 | 1.00 | 0.43 | 0.91 | 3.6E-04 | 0.00 | 0.22 | 3.9E-04 | 0.00 | 0.94 | 0.00 | 0.00 | 7.02 |
| p_Proteobacteria  c_Alphaproteobacteria | 0.97 | 1.00 | 0.52 | 0.91 | 2.0E-04 | 0.00 | 1.83 | 3.9E-04 | 0.00 | 0.47 | 0.02 | 0.00 | 0.65 |
| p_Elusimicrobia  c_Elusimicrobia | 0.69 | 1.00 | 0.58 | 0.91 | 0.00 | 0.00 | 0.44 | 1.7E-04 | 0.00 | 0.52 | 0.00 | 0.00 | 0.42 |
| p_Fusobacteria  c_Fusobacteriia | 1.00 | 1.00 | 0.75 | 0.91 | 0.00 | 0.00 | 0.00 | 0.00 | 0.00 | 0.00 | 0.00 | 0.00 | 0.09 |
| p_Proteobacteria  c_Epsilonproteobacteria | 0.63 | 1.00 | 0.25 | 0.91 | 0.00 | 0.00 | 0.35 | 0.00 | 0.00 | 0.12 | 0.00 | 0.00 | 0.02 |
| p_TM7  c_UC TM7 | 0.63 | 1.00 | 0.88 | 0.91 | 0.00 | 0.00 | 0.30 | 0.00 | 0.00 | 0.18 | 0.00 | 0.00 | 0.16 |
| p_Verrucomicrobia  c_Opitutae | 0.22 | 0.80 | 0.44 | 0.91 | 0.00 | 0.00 | 0.09 | 0.00 | 0.00 | 0.01 | 0.00 | 0.00 | 0.33 |
| **Order** | | | | | | | | | | | | | |
| p_Bacteroidetes  c_Bacteroidia  o_Bacteroidales | 0.76 | 1.00 | 0.34 | 0.97 | 39.11 | 3.58 | 76.23 | 42.17 | 16.10 | 67.72 | 50.38 | 11.40 | 77.79 |
| p_Firmicutes  c_Clostridia  o_Clostridiales | 0.43 | 0.99 | 0.45 | 0.97 | 32.66 | 9.57 | 43.83 | 32.79 | 15.75 | 54.09 | 25.80 | 15.66 | 59.34 |
| p_Firmicutes  c_Negativicutes  o_Selenomonadales | 0.86 | 1.00 | 0.79 | 0.97 | 8.89 | 3.33 | 28.32 | 8.18 | 3.44 | 29.86 | 6.80 | 1.33 | 34.83 |
| p_Proteobacteria  c_Gammaproteobacteria  o_Enterobacteriales | 0.63 | 0.99 | 0.04 | 0.63 | 2.50 | 0.05 | 23.39 | 1.49 | 0.10 | 23.46 | 0.39 | 0.05 | 22.78 |
| p_Actinobacteria  c_Actinobacteria  o_Bifidobacteriales | 0.04 | 0.64 | 0.41 | 0.97 | 1.51 | 0.48 | 16.20 | 0.74 | 0.18 | 6.93 | 0.63 | 0.07 | 4.05 |
| p_Firmicutes  c_Erysipelotrichia  o_Erysipelotrichales | 0.17 | 0.80 | 0.89 | 0.97 | 0.69 | 0.03 | 1.90 | 0.74 | 0.02 | 1.59 | 0.54 | 0.06 | 3.75 |
| p_Firmicutes  c_Bacilli  o_Lactobacillales | 1.00 | 1.00 | 0.34 | 0.97 | 0.41 | 0.01 | 6.26 | 0.88 | 0.03 | 6.61 | 0.42 | 0.04 | 2.14 |
| p_Actinobacteria  c_Coriobacteriia  o_Coriobacteriales | 0.06 | 0.64 | 0.59 | 0.97 | 0.27 | 0.03 | 4.22 | 0.20 | 0.04 | 1.12 | 0.07 | 0.04 | 2.62 |
| p_Proteobacteria  c_Betaproteobacteria  o_Burkholderiales | 0.38 | 0.99 | 0.50 | 0.97 | 0.19 | 0.01 | 0.70 | 0.10 | 0.01 | 2.05 | 0.14 | 0.01 | 1.07 |
| p_Proteobacteria  c_Gammaproteobacteria  o_UC Gammaproteobacteria | 0.14 | 0.80 | 0.38 | 0.97 | 0.17 | 1.6E-03 | 1.58 | 0.06 | 3.2E-04 | 2.36 | 0.02 | 7.0E-04 | 0.97 |
| p_Proteobacteria  c_Gammaproteobacteria  o_Pasteurellales | 0.22 | 0.80 | 0.41 | 0.97 | 0.13 | 0.01 | 4.60 | 0.05 | 3.8E-04 | 0.48 | 0.14 | 1.3E-03 | 1.77 |
| p_Tenericutes  c_Mollicutes  o_RF9 | 0.81 | 1.00 | 0.50 | 0.97 | 0.09 | 4.4E-04 | 7.00 | 0.12 | 1.2E-03 | 5.93 | 0.24 | 8.4E-04 | 7.92 |
| p_Proteobacteria  c_Deltaproteobacteria  o_Desulfovibrionales | 0.24 | 0.80 | 0.62 | 0.97 | 0.04 | 0.00 | 0.98 | 0.06 | 0.00 | 0.76 | 0.04 | 0.00 | 0.39 |
| p_Proteobacteria  c_Gammaproteobacteria  o_Aeromonadales | 0.63 | 0.99 | 0.05 | 0.63 | 0.02 | 7.0E-04 | 8.42 | 0.31 | 9.7E-04 | 16.67 | 0.11 | 1.1E-03 | 8.42 |
| p_Firmicutes  c_UC Firmicutes  o_UC Firmicutes | 0.36 | 0.99 | 0.79 | 0.97 | 0.01 | 0.00 | 1.95 | 4.5E-03 | 4.0E-04 | 0.67 | 0.01 | 0.00 | 0.25 |
| p_Proteobacteria  c_Gammaproteobacteria  o_B38 | 1.00 | 1.00 | 0.06 | 0.63 | 0.01 | 0.00 | 0.44 | 0.01 | 0.00 | 0.22 | 1.9E-03 | 0.00 | 0.03 |
| p_Cyanobacteria  c_Melainabacteria  o_Gastranaerophilales | 0.64 | 0.99 | 0.85 | 0.97 | 4.4E-03 | 0.00 | 1.51 | 1.4E-03 | 0.00 | 0.57 | 3.3E-03 | 0.00 | 4.04 |
| p_Actinobacteria  c_Actinobacteria  o_Micrococcales | 0.67 | 0.99 | 0.54 | 0.97 | 3.8E-03 | 0.00 | 0.02 | 2.6E-03 | 4.0E-04 | 0.03 | 2.0E-03 | 3.5E-04 | 0.02 |
| p_Lentisphaerae  c_Lentisphaeria  o_Victivallales | 0.50 | 0.99 | 0.91 | 0.97 | 1.5E-03 | 0.00 | 0.19 | 1.1E-03 | 0.00 | 0.01 | 0.00 | 0.00 | 0.10 |
| p_Verrucomicrobia  c_Verrucomicrobiae  o_Verrucomicrobiales | 0.21 | 0.80 | 0.73 | 0.97 | 8.4E-04 | 0.00 | 1.38 | 1.7E-04 | 0.00 | 0.73 | 0.00 | 0.00 | 0.02 |
| p_Tenericutes  c_Mollicutes  o_NB1-n | 0.19 | 0.80 | 0.50 | 0.97 | 5.6E-04 | 0.00 | 0.29 | 9.2E-04 | 0.00 | 0.20 | 0.00 | 0.00 | 1.80 |
| p_Proteobacteria  c_UC Proteobacteria  o_UC Proteobacteria | 0.03 | 0.64 | 0.49 | 0.97 | 4.4E-04 | 0.00 | 0.04 | 3.0E-03 | 0.00 | 0.08 | 0.00 | 0.00 | 0.09 |
| p_Spirochaetae  c_Spirochaetes  o_Spirochaetales | 0.90 | 1.00 | 0.43 | 0.97 | 3.6E-04 | 0.00 | 0.22 | 3.9E-04 | 0.00 | 0.94 | 0.00 | 0.00 | 7.02 |
| p_Proteobacteria  c_Alphaproteobacteria  o_Rhodospirillales | 0.97 | 1.00 | 0.85 | 0.97 | 2.0E-04 | 0.00 | 1.76 | 3.9E-04 | 0.00 | 0.47 | 3.4E-03 | 0.00 | 0.65 |
| p_Elusimicrobia  c_Elusimicrobia  o_Elusimicrobiales | 0.69 | 0.99 | 0.58 | 0.97 | 0.00 | 0.00 | 0.44 | 1.7E-04 | 0.00 | 0.52 | 0.00 | 0.00 | 0.42 |
| p_Fusobacteria  c_Fusobacteriia  o_Fusobacteriales | 1.00 | 1.00 | 0.75 | 0.97 | 0.00 | 0.00 | 2.7E-03 | 0.00 | 0.00 | 2.4E-03 | 0.00 | 0.00 | 0.09 |
| p_Proteobacteria  c_Alphaproteobacteria  o_UC Alphaproteobacteria | 0.50 | 0.99 | 1.00 | 1.00 | 0.00 | 0.00 | 0.41 | 0.00 | 0.00 | 1.6E-03 | 0.00 | 0.00 | 0.06 |
| p_Proteobacteria  c_Betaproteobacteria  o_Neisseriales | 0.54 | 0.99 | 0.63 | 0.97 | 0.00 | 0.00 | 0.18 | 0.00 | 0.00 | 1.63 | 0.00 | 0.00 | 1.76 |
| p_Proteobacteria  c_Betaproteobacteria  o_UC Betaproteobacteria | 1.00 | 1.00 | 1.00 | 1.00 | 0.00 | 0.00 | 0.06 | 1.6E-04 | 0.00 | 0.06 | 0.00 | 0.00 | 0.06 |
| p_Proteobacteria  c_Epsilonproteobacteria  o_Campylobacterales | 0.63 | 0.99 | 0.25 | 0.97 | 0.00 | 0.00 | 0.35 | 0.00 | 0.00 | 0.12 | 0.00 | 0.00 | 0.02 |
| p_TM7  c_UC TM7  o_UC TM7 | 0.63 | 0.99 | 0.88 | 0.97 | 0.00 | 0.00 | 0.30 | 0.00 | 0.00 | 0.18 | 0.00 | 0.00 | 0.16 |
| p_Tenericutes  c_Mollicutes  o_Anaeroplasmatales | 0.84 | 1.00 | 0.84 | 0.97 | 0.00 | 0.00 | 0.03 | 5.8E-04 | 0.00 | 0.02 | 3.9E-04 | 0.00 | 1.11 |
| p_Verrucomicrobia  c_Opitutae  o_vadinHA64 | 0.22 | 0.80 | 0.44 | 0.97 | 0.00 | 0.00 | 0.09 | 0.00 | 0.00 | 0.01 | 0.00 | 0.00 | 0.33 |
| **Family** | | | | | | | | | | | | | |
| p_Bacteroidetes  c_Bacteroidia  o_Bacteroidales  f_Prevotellaceae | 0.54 | 0.90 | 0.03 | 0.63 | 26.76 | 0.04 | 72.61 | 23.16 | 0.03 | 63.80 | 36.53 | 0.07 | 4.05 |
| p_Firmicutes  c_Clostridia  o_Clostridiales  f_Ruminococcaceae | 0.71 | 0.91 | 0.31 | 0.97 | 20.21 | 4.50 | 31.18 | 16.46 | 6.89 | 35.36 | 13.45 | 6.83 | 0.02 |
| p_Firmicutes  c_Clostridia  o_Clostridiales  f_Lachnospiraceae | 0.09 | 0.60 | 0.41 | 0.97 | 9.56 | 4.62 | 18.91 | 12.64 | 6.16 | 36.01 | 10.82 | 6.55 | 2.62 |
| p_Firmicutes  c_Negativicutes  o_Selenomonadales  f_Veillonellaceae | 0.95 | 1.00 | 0.74 | 0.97 | 8.68 | 2.65 | 23.67 | 6.90 | 1.06 | 29.72 | 6.69 | 1.31 | 41.51 |
| p_Proteobacteria  c_Gammaproteobacteria  o_Enterobacteriales  f_Enterobacteriaceae | 0.63 | 0.90 | 0.04 | 0.63 | 2.50 | 0.05 | 23.39 | 1.49 | 0.10 | 23.46 | 0.39 | 0.05 | 8.46 |
| p_Actinobacteria  c_Actinobacteria  o_Bifidobacteriales  f_Bifidobacteriaceae | 0.04 | 0.60 | 0.73 | 0.97 | 1.51 | 0.48 | 16.20 | 0.74 | 0.18 | 6.93 | 0.63 | 0.07 | 69.11 |
| p_Bacteroidetes  c_Bacteroidia  o_Bacteroidales  f_Porphyromonadaceae | 0.43 | 0.84 | 0.41 | 0.97 | 1.28 | 4.8E-03 | 4.12 | 1.88 | 0.01 | 8.66 | 1.05 | 0.01 | 5.12 |
| p_Bacteroidetes  c_Bacteroidia  o_Bacteroidales  f_Bacteroidaceae | 0.15 | 0.60 | 0.50 | 0.97 | 1.00 | 0.05 | 28.14 | 1.60 | 0.11 | 32.14 | 0.48 | 0.04 | 0.97 |
| p_Firmicutes  c_Erysipelotrichia  o_Erysipelotrichales  f_Erysipelotrichaceae | 0.17 | 0.60 | 0.89 | 0.98 | 0.69 | 0.03 | 1.90 | 0.74 | 0.02 | 1.59 | 0.54 | 0.06 | 10.00 |
| p_Bacteroidetes  c_Bacteroidia  o_Bacteroidales  f_UC Bacteroidales | 0.12 | 0.60 | 0.79 | 0.97 | 0.43 | 0.03 | 5.61 | 0.49 | 0.05 | 12.07 | 0.43 | 0.03 | 4.04 |
| p_Bacteroidetes  c_Bacteroidia  o_Bacteroidales  f_Rikenellaceae | 0.22 | 0.60 | 0.59 | 0.97 | 0.36 | 3.7E-04 | 2.40 | 0.72 | 0.01 | 5.86 | 0.59 | 2.2E-03 | 0.42 |
| p_Firmicutes  c_Clostridia  o_Clostridiales  f_UC Clostridiales | 0.09 | 0.60 | 0.68 | 0.97 | 0.31 | 0.10 | 3.13 | 0.23 | 0.07 | 2.98 | 0.32 | 0.05 | 0.02 |
| p_Actinobacteria  c_Coriobacteriia  o_Coriobacteriales  f_Coriobacteriaceae | 0.06 | 0.60 | 0.59 | 0.97 | 0.27 | 0.03 | 4.22 | 0.20 | 0.04 | 1.12 | 0.07 | 0.04 | 1.02 |
| p_Firmicutes  c_Bacilli  o_Lactobacillales  f_Lactobacillaceae | 0.12 | 0.60 | 0.24 | 0.97 | 0.22 | 3.0E-03 | 5.81 | 0.15 | 1.6E-03 | 1.74 | 0.10 | 2.8E-03 | 0.01 |
| p_Proteobacteria  c_Gammaproteobacteria  o_UC Gammaproteobacteria  f_UC Gammaproteobacteria | 0.14 | 0.60 | 0.38 | 0.97 | 0.17 | 1.6E-03 | 1.58 | 0.06 | 3.2E-04 | 2.36 | 0.02 | 7.0E-04 | 2.11 |
| p_Firmicutes  c_Negativicutes  o_Selenomonadales  f_UC Selenomonadales | 0.46 | 0.88 | 0.79 | 0.97 | 0.16 | 7.0E-04 | 4.66 | 0.15 | 0.00 | 5.05 | 0.34 | 6.5E-04 | 1.36 |
| p_Proteobacteria  c_Betaproteobacteria  o_Burkholderiales  f_Alcaligenaceae | 0.19 | 0.60 | 0.31 | 0.97 | 0.16 | 0.01 | 0.70 | 0.10 | 0.01 | 0.99 | 0.14 | 0.01 | 1.00 |
| p_Proteobacteria  c_Gammaproteobacteria  o_Pasteurellales  f_Pasteurellaceae | 0.22 | 0.60 | 0.41 | 0.97 | 0.13 | 0.01 | 4.60 | 0.05 | 3.8E-04 | 0.48 | 0.14 | 1.3E-03 | 0.13 |
| p_Firmicutes  c_Clostridia  o_Clostridiales  f_Christensenellaceae | 0.17 | 0.60 | 0.74 | 0.97 | 0.11 | 0.00 | 1.28 | 0.12 | 4.2E-04 | 2.51 | 0.17 | 5.4E-04 | 0.10 |
| p_Firmicutes  c_Clostridia  o_Clostridiales  f_Peptostreptococcaceae | 0.10 | 0.60 | 0.31 | 0.97 | 0.10 | 3.8E-04 | 0.64 | 0.06 | 0.00 | 0.20 | 0.10 | 0.00 | 21.47 |
| p_Bacteroidetes  c_Bacteroidia  o_Bacteroidales  f_S24-7 | 0.76 | 0.91 | 0.84 | 0.97 | 0.10 | 0.00 | 9.30 | 0.15 | 0.00 | 2.56 | 0.04 | 0.00 | 0.04 |
| p_Tenericutes  c_Mollicutes  o_RF9  f_UC RF9 | 0.81 | 0.95 | 0.50 | 0.97 | 0.09 | 4.4E-04 | 7.00 | 0.12 | 1.2E-03 | 5.93 | 0.24 | 8.4E-04 | 1.08 |
| p_Firmicutes  c_Clostridia  o_Clostridiales  f_vadinBB60 | 0.08 | 0.60 | 0.32 | 0.97 | 0.09 | 0.00 | 0.95 | 0.02 | 0.00 | 0.26 | 0.02 | 0.00 | 34.85 |
| p_Firmicutes  c_Clostridia  o_Clostridiales  f_Clostridiaceae 1 | 0.39 | 0.83 | 0.45 | 0.97 | 0.06 | 1.5E-03 | 0.84 | 0.02 | 0.00 | 0.63 | 0.14 | 0.00 | 1.93 |
| p_Proteobacteria  c_Deltaproteobacteria  o_Desulfovibrionales  f_Desulfovibrionaceae | 0.24 | 0.64 | 0.62 | 0.97 | 0.04 | 0.00 | 0.98 | 0.06 | 0.00 | 0.76 | 0.04 | 0.00 | 1.06 |
| p_Firmicutes  c_Bacilli  o_Lactobacillales  f_Streptococcaceae | 0.06 | 0.60 | 0.27 | 0.97 | 0.04 | 0.01 | 5.65 | 0.10 | 0.01 | 6.52 | 0.07 | 3.8E-03 | 3.75 |
| p_Firmicutes  c_Clostridia  o_Clostridiales  f_Defluviitaleaceae | 0.54 | 0.90 | 0.59 | 0.97 | 0.03 | 3.7E-04 | 0.52 | 0.04 | 0.01 | 0.31 | 0.03 | 0.02 | 1.15 |
| p_Proteobacteria  c_Gammaproteobacteria  o_Aeromonadales  f_Succinivibrionaceae | 0.63 | 0.90 | 0.05 | 0.63 | 0.02 | 7.0E-04 | 8.42 | 0.31 | 9.7E-04 | 16.67 | 0.11 | 1.1E-03 | 3.62 |
| p_Firmicutes  c_Clostridia  o_Clostridiales  f_Family XIII | 0.38 | 0.83 | 0.68 | 0.97 | 0.02 | 0.00 | 0.15 | 0.05 | 0.00 | 0.18 | 0.03 | 0.00 | 34.83 |
| p_Firmicutes  c_UC Firmicutes  o_UC Firmicutes  f_UC Firmicutes | 0.36 | 0.83 | 0.79 | 0.97 | 0.01 | 0.00 | 1.95 | 4.5E-03 | 4.0E-04 | 0.67 | 0.01 | 0.00 | 0.25 |
| p_Proteobacteria  c_Gammaproteobacteria  o_B38  f_UC B38 | 1.00 | 1.00 | 0.06 | 0.63 | 0.01 | 0.00 | 0.44 | 0.01 | 0.00 | 0.22 | 1.9E-03 | 0.00 | 0.09 |
| p_Proteobacteria  c_Betaproteobacteria  o_Burkholderiales  f_Comamonadaceae | 0.68 | 0.90 | 0.36 | 0.97 | 0.01 | 0.00 | 0.18 | 7.1E-04 | 0.00 | 1.53 | 0.00 | 0.00 | 0.10 |
| p_Cyanobacteria  c_Melainabacteria  o_Gastranaerophilales  f_UC Gastranaerophilales | 0.64 | 0.90 | 0.85 | 0.97 | 4.4E-03 | 0.00 | 1.51 | 1.4E-03 | 0.00 | 0.57 | 3.3E-03 | 0.00 | 0.65 |
| p_Actinobacteria  c_Actinobacteria  o_Micrococcales  f_Micrococcaceae | 0.67 | 0.90 | 0.54 | 0.97 | 3.8E-03 | 0.00 | 0.02 | 2.6E-03 | 4.0E-04 | 0.03 | 2.0E-03 | 3.5E-04 | 0.06 |
| p_Firmicutes  c_Clostridia  o_Clostridiales  f_Peptococcaceae | 0.64 | 0.90 | 0.36 | 0.97 | 3.5E-03 | 0.00 | 0.09 | 4.7E-03 | 0.00 | 0.08 | 3.4E-03 | 0.00 | 1.07 |
| p_Lentisphaerae  c_Lentisphaeria  o_Victivallales  f_Victivallaceae | 0.50 | 0.89 | 0.91 | 0.98 | 1.5E-03 | 0.00 | 0.19 | 1.1E-03 | 0.00 | 0.01 | 0.00 | 0.00 | 0.58 |
| p_Firmicutes  c_Negativicutes  o_Selenomonadales  f_Acidaminococcaceae | 0.33 | 0.81 | 0.59 | 0.97 | 1.2E-03 | 0.00 | 1.56 | 4.4E-03 | 0.00 | 3.24 | 4.6E-03 | 0.00 | 4.8E-04 |
| p_Firmicutes  c_Bacilli  o_Lactobacillales  f_Leuconostocaceae | 0.41 | 0.84 | 0.38 | 0.97 | 1.0E-03 | 0.00 | 0.02 | 4.1E-04 | 0.00 | 0.02 | 0.00 | 0.00 | 1.76 |
| p_Verrucomicrobia  c_Verrucomicrobiae  o_Verrucomicrobiales  f_Verrucomicrobiaceae | 0.21 | 0.60 | 0.73 | 0.97 | 8.4E-04 | 0.00 | 1.38 | 1.7E-04 | 0.00 | 0.73 | 0.00 | 0.00 | 0.06 |
| p_Firmicutes  c_Bacilli  o_Lactobacillales  f_Enterococcaceae | 0.76 | 0.91 | 0.50 | 0.97 | 5.7E-04 | 0.00 | 0.01 | 1.7E-04 | 0.00 | 1.12 | 0.00 | 0.00 | 0.39 |
| p_Tenericutes  c_Mollicutes  o_NB1-n  f_UC NB1-n | 0.19 | 0.60 | 0.50 | 0.97 | 5.6E-04 | 0.00 | 0.29 | 9.2E-04 | 0.00 | 0.20 | 0.00 | 0.00 | 0.02 |
| p_Proteobacteria  c_UC Proteobacteria  o_UC Proteobacteria  f_UC Proteobacteria | 0.03 | 0.60 | 0.01 | 0.54 | 4.4E-04 | 0.00 | 0.04 | 3.0E-03 | 0.00 | 0.08 | 0.00 | 0.00 | 8.42 |
| p_Spirochaetae  c_Spirochaetes  o_Spirochaetales  f_Spirochaetaceae | 0.90 | 1.00 | 0.43 | 0.97 | 3.6E-04 | 0.00 | 0.22 | 3.9E-04 | 0.00 | 0.90 | 0.00 | 0.00 | 0.03 |
| p_Proteobacteria  c_Alphaproteobacteria  o_Rhodospirillales  f_Rhodospirillaceae | 0.97 | 1.00 | 0.85 | 0.97 | 2.0E-04 | 0.00 | 1.76 | 3.9E-04 | 0.00 | 0.47 | 3.4E-03 | 0.00 | 22.78 |
| p_Elusimicrobia  c_Elusimicrobia  o_Elusimicrobiales  f_Elusimicrobiaceae | 0.69 | 0.90 | 0.58 | 0.97 | 0.00 | 0.00 | 0.44 | 1.7E-04 | 0.00 | 0.52 | 0.00 | 0.00 | 1.77 |
| p_Fusobacteria  c_Fusobacteriia  o_Fusobacteriales  f_Fusobacteriaceae | 1.00 | 1.00 | 0.75 | 0.97 | 0.00 | 0.00 | 2.7E-03 | 0.00 | 0.00 | 2.4E-03 | 0.00 | 0.00 | 0.97 |
| p_Proteobacteria  c_Alphaproteobacteria  o_UC Alphaproteobacteria  f_UC Alphaproteobacteria | 0.50 | 0.89 | 1.00 | 1.00 | 0.00 | 0.00 | 0.41 | 0.00 | 0.00 | 1.6E-03 | 0.00 | 0.00 | 0.09 |
| p_Proteobacteria  c_Betaproteobacteria  o_Burkholderiales  f_Oxalobacteraceae | 0.75 | 0.91 | 1.00 | 1.00 | 0.00 | 0.00 | 4.4E-04 | 0.00 | 0.00 | 0.07 | 0.00 | 0.00 | 0.34 |
| p_Proteobacteria  c_Betaproteobacteria  o_Neisseriales  f_Neisseriaceae | 0.38 | 0.83 | 0.63 | 0.97 | 0.00 | 0.00 | 0.18 | 0.00 | 0.00 | 1.63 | 0.00 | 0.00 | 6.69 |
| p_Proteobacteria  c_Betaproteobacteria  o_UC Betaproteobacteria  f_UC Betaproteobacteria | 1.00 | 1.00 | 1.00 | 1.00 | 0.00 | 0.00 | 0.06 | 1.6E-04 | 0.00 | 0.06 | 0.00 | 0.00 | 0.16 |
| p_Proteobacteria  c_Epsilonproteobacteria  o_Campylobacterales  f_Campylobacteraceae | 0.63 | 0.90 | 0.25 | 0.97 | 0.00 | 0.00 | 0.35 | 0.00 | 0.00 | 0.12 | 0.00 | 0.00 | 1.11 |
| p_Spirochaetae  c_Spirochaetes  o_Spirochaetales  f_Brachyspiraceae | 1.00 | 1.00 | 1.00 | 1.00 | 0.00 | 0.00 | 0.01 | 0.00 | 0.00 | 0.04 | 0.00 | 0.00 | 1.80 |
| p_TM7  c_UC TM7  o_UC TM7  f_UC TM7 | 0.63 | 0.90 | 0.88 | 0.98 | 0.00 | 0.00 | 0.30 | 0.00 | 0.00 | 0.18 | 0.00 | 0.00 | 7.92 |
| p_Tenericutes  c_Mollicutes  o_Anaeroplasmatales  f_Anaeroplasmataceae | 0.84 | 0.97 | 0.84 | 0.97 | 0.00 | 0.00 | 0.03 | 5.8E-04 | 0.00 | 0.02 | 3.9E-04 | 0.00 | 0.33 |
| p_Verrucomicrobia  c_Opitutae  o_vadinHA64  f_UC vadinHA64 | 0.22 | 0.60 | 0.44 | 0.97 | 0.00 | 0.00 | 0.09 | 0.00 | 0.00 | 0.01 | 0.00 | 0.00 | 0.02 |
| **Genus** | **p value** | **FDR** | **p value** | **FDR** | **Median** | **Min** | **Max** | **Median** | **Min** | **Max** | **Median** | **Min** | **Max** |
| p_Bacteroidetes  c_Bacteroidia  o_Bacteroidales  f_Prevotellaceae  g_Prevotella | 0.39 | 0.82 | 0.03 | 0.77 | 24.98 | 0.04 | 64.58 | 21.72 | 0.03 | 54.69 | 35.04 | 0.07 | 62.67 |
| p_Firmicutes  c_Clostridia  o_Clostridiales  f_Ruminococcaceae  g_Faecalibacterium | 0.27 | 0.82 | 0.17 | 1.00 | 6.49 | 1.04 | 25.47 | 6.53 | 3.63 | 30.01 | 7.02 | 2.01 | 10.61 |
| p_Firmicutes  c_Clostridia  o_Clostridiales  f_Ruminococcaceae  g_UC Ruminococcaceae | 0.33 | 0.82 | 0.79 | 1.00 | 5.03 | 0.51 | 18.76 | 5.41 | 0.60 | 20.25 | 4.19 | 0.65 | 17.08 |
| p_Firmicutes  c_Negativicutes  o_Selenomonadales  f_Veillonellaceae  g_Dialister | 1.00 | 1.00 | 0.45 | 1.00 | 4.50 | 1.11 | 14.25 | 4.23 | 0.30 | 20.66 | 5.78 | 0.99 | 13.64 |
| p_Firmicutes  c_Clostridia  o_Clostridiales  f_Lachnospiraceae  g_UC Lachnospiraceae | 0.14 | 0.67 | 0.45 | 1.00 | 4.41 | 1.48 | 10.78 | 6.61 | 2.78 | 28.73 | 4.43 | 2.08 | 15.22 |
| p_Firmicutes  c_Clostridia  o_Clostridiales  f_Lachnospiraceae  g_Blautia | 0.33 | 0.82 | 0.89 | 1.00 | 1.55 | 0.21 | 3.81 | 1.13 | 0.36 | 10.50 | 0.91 | 0.10 | 6.12 |
| p_Actinobacteria  c_Actinobacteria  o_Bifidobacteriales  f_Bifidobacteriaceae  g_Bifidobacterium | 0.04 | 0.67 | 0.41 | 1.00 | 1.51 | 0.48 | 16.20 | 0.74 | 0.18 | 6.93 | 0.63 | 0.07 | 4.05 |
| p_Bacteroidetes  c_Bacteroidia  o_Bacteroidales  f_Prevotellaceae  g_Alloprevotella | 0.30 | 0.82 | 0.31 | 1.00 | 1.30 | 1.7E-03 | 6.80 | 1.88 | 0.00 | 7.44 | 0.79 | 1.6E-03 | 8.91 |
| p_Firmicutes  c_Negativicutes  o_Selenomonadales  f_Veillonellaceae  g_Megasphaera | 0.09 | 0.67 | 0.79 | 1.00 | 1.07 | 1.0E-03 | 15.07 | 0.37 | 0.00 | 8.34 | 0.56 | 2.6E-03 | 5.45 |
| p_Bacteroidetes  c_Bacteroidia  o_Bacteroidales  f_Bacteroidaceae  g_Bacteroides | 0.15 | 0.69 | 0.50 | 1.00 | 1.00 | 0.05 | 28.14 | 1.60 | 0.11 | 32.14 | 0.48 | 0.04 | 41.51 |
| p_Firmicutes  c_Clostridia  o_Clostridiales  f_Lachnospiraceae  g_Incertae Sedis | 0.33 | 0.82 | 0.84 | 1.00 | 0.91 | 0.36 | 3.31 | 0.93 | 0.53 | 2.23 | 0.91 | 0.42 | 3.75 |
| p_Firmicutes  c_Clostridia  o_Clostridiales  f_Ruminococcaceae  g_Incertae Sedis | 0.71 | 0.95 | 0.79 | 1.00 | 0.65 | 0.08 | 2.95 | 0.57 | 0.16 | 3.44 | 0.70 | 0.06 | 3.52 |
| p_Bacteroidetes  c_Bacteroidia  o_Bacteroidales  f_Porphyromonadaceae  g_UC Porphyromonadaceae | 0.95 | 1.00 | 0.89 | 1.00 | 0.52 | 1.3E-03 | 3.98 | 0.57 | 1.2E-03 | 8.60 | 0.47 | 2.2E-03 | 8.33 |
| p_Firmicutes  c_Clostridia  o_Clostridiales  f_Ruminococcaceae  g_Ruminococcus | 0.10 | 0.67 | 0.45 | 1.00 | 0.51 | 1.8E-03 | 3.11 | 0.45 | 0.01 | 6.27 | 0.49 | 0.01 | 4.33 |
| p_Proteobacteria  c_Gammaproteobacteria  o_Enterobacteriales  f_Enterobacteriaceae  g_Escherichia-Shigella | 1.00 | 1.00 | 0.74 | 1.00 | 0.44 | 0.05 | 17.76 | 0.31 | 0.01 | 15.07 | 0.21 | 0.02 | 21.87 |
| p_Bacteroidetes  c_Bacteroidia  o_Bacteroidales  f_UC Bacteroidales  g_UC Bacteroidales | 0.12 | 0.67 | 0.79 | 1.00 | 0.43 | 0.03 | 5.61 | 0.49 | 0.05 | 12.07 | 0.43 | 0.03 | 10.00 |
| p_Firmicutes  c_Clostridia  o_Clostridiales  f_Lachnospiraceae  g_Roseburia | 0.36 | 0.82 | 0.54 | 1.00 | 0.42 | 0.13 | 1.25 | 0.99 | 0.05 | 2.20 | 0.45 | 0.28 | 4.12 |
| p_Proteobacteria  c_Gammaproteobacteria  o_Enterobacteriales  f_Enterobacteriaceae  g_Klebsiella | 0.27 | 0.82 | 0.08 | 0.99 | 0.41 | 1.2E-03 | 2.91 | 0.08 | 0.00 | 6.56 | 0.02 | 4.5E-04 | 0.22 |
| p_Proteobacteria  c_Gammaproteobacteria  o_Enterobacteriales  f_Enterobacteriaceae  g_UC Enterobacteriaceae | 0.33 | 0.82 | 0.02 | 0.77 | 0.34 | 1.6E-03 | 4.29 | 0.11 | 0.01 | 4.08 | 0.09 | 3.2E-03 | 0.55 |
| p_Firmicutes  c_Erysipelotrichia  o_Erysipelotrichales  f_Erysipelotrichaceae  g_Catenibacterium | 0.71 | 0.95 | 0.64 | 1.00 | 0.34 | 4.4E-04 | 1.81 | 0.36 | 7.9E-04 | 1.26 | 0.16 | 4.2E-04 | 3.09 |
| p_Firmicutes  c_Clostridia  o_Clostridiales  f_UC Clostridiales  g_UC Clostridiales | 0.09 | 0.67 | 0.68 | 1.00 | 0.31 | 0.10 | 3.13 | 0.23 | 0.07 | 2.98 | 0.32 | 0.05 | 1.93 |
| p_Bacteroidetes  c_Bacteroidia  o_Bacteroidales  f_Porphyromonadaceae  g_Parabacteroides | 0.54 | 0.88 | 0.64 | 1.00 | 0.30 | 1.8E-03 | 0.92 | 0.21 | 0.01 | 4.21 | 0.10 | 2.4E-03 | 1.04 |
| p_Firmicutes  c_Clostridia  o_Clostridiales  f_Lachnospiraceae  g_Lachnospira | 0.46 | 0.85 | 0.50 | 1.00 | 0.24 | 0.03 | 4.07 | 0.52 | 2.3E-03 | 2.18 | 0.20 | 0.01 | 3.64 |
| p_Firmicutes  c_Bacilli  o_Lactobacillales  f_Lactobacillaceae  g_Lactobacillus | 0.12 | 0.67 | 0.24 | 1.00 | 0.22 | 3.0E-03 | 5.81 | 0.15 | 1.6E-03 | 1.74 | 0.10 | 2.8E-03 | 1.02 |
| p_Proteobacteria  c_Gammaproteobacteria  o_Enterobacteriales  f_Enterobacteriaceae  g_Enterobacter | 0.81 | 0.99 | 0.02 | 0.77 | 0.21 | 0.00 | 5.39 | 0.14 | 3.8E-03 | 2.43 | 0.02 | 4.5E-04 | 0.46 |
| p_Proteobacteria  c_Gammaproteobacteria  o_UC Gammaproteobacteria  f_UC Gammaproteobacteria  g_UC Gammaproteobacteria | 0.14 | 0.67 | 0.38 | 1.00 | 0.17 | 1.6E-03 | 1.58 | 0.06 | 3.2E-04 | 2.36 | 0.02 | 7.0E-04 | 0.97 |
| p_Bacteroidetes  c_Bacteroidia  o_Bacteroidales  f_Rikenellaceae  g_Alistipes | 0.50 | 0.85 | 0.89 | 1.00 | 0.17 | 3.7E-04 | 1.80 | 0.06 | 4.5E-03 | 3.10 | 0.04 | 2.2E-03 | 1.75 |
| p_Firmicutes  c_Negativicutes  o_Selenomonadales  f_UC Selenomonadales  g_UC Selenomonadales | 0.46 | 0.85 | 0.79 | 1.00 | 0.16 | 7.0E-04 | 4.66 | 0.15 | 0.00 | 5.05 | 0.34 | 6.5E-04 | 3.62 |
| p_Proteobacteria  c_Betaproteobacteria  o_Burkholderiales  f_Alcaligenaceae  g_Sutterella | 0.43 | 0.83 | 0.22 | 1.00 | 0.13 | 0.00 | 0.52 | 0.05 | 1.2E-03 | 0.50 | 0.12 | 4.4E-03 | 0.69 |
| p_Firmicutes  c_Clostridia  o_Clostridiales  f_Lachnospiraceae  g_Pseudobutyrivibrio | 0.46 | 0.85 | 0.79 | 1.00 | 0.13 | 2.6E-03 | 0.82 | 0.24 | 3.5E-03 | 2.71 | 0.08 | 0.02 | 2.50 |
| p_Proteobacteria  c_Gammaproteobacteria  o_Pasteurellales  f_Pasteurellaceae  g_Haemophilus | 0.22 | 0.76 | 0.38 | 1.00 | 0.12 | 0.01 | 4.60 | 0.05 | 3.8E-04 | 0.47 | 0.13 | 1.3E-03 | 1.75 |
| p_Firmicutes  c_Clostridia  o_Clostridiales  f_Christensenellaceae  g_UC Christensenellaceae | 0.15 | 0.69 | 0.74 | 1.00 | 0.11 | 0.00 | 1.28 | 0.12 | 4.2E-04 | 2.48 | 0.17 | 5.4E-04 | 1.36 |
| p_Actinobacteria  c_Coriobacteriia  o_Coriobacteriales  f_Coriobacteriaceae  g_Collinsella | 0.14 | 0.67 | 1.00 | 1.00 | 0.10 | 0.02 | 0.58 | 0.06 | 3.8E-04 | 0.25 | 0.05 | 0.01 | 0.32 |
| p_Firmicutes  c_Clostridia  o_Clostridiales  f_Peptostreptococcaceae  g_Incertae Sedis | 0.10 | 0.67 | 0.31 | 1.00 | 0.10 | 3.8E-04 | 0.64 | 0.06 | 0.00 | 0.20 | 0.10 | 0.00 | 1.08 |
| p_Bacteroidetes  c_Bacteroidia  o_Bacteroidales  f_S24-7  g_UC S24-7 | 0.76 | 0.98 | 0.84 | 1.00 | 0.10 | 0.00 | 9.30 | 0.15 | 0.00 | 2.56 | 0.04 | 0.00 | 0.97 |
| p_Firmicutes  c_Erysipelotrichia  o_Erysipelotrichales  f_Erysipelotrichaceae  g_Incertae Sedis | 0.71 | 0.95 | 0.70 | 1.00 | 0.10 | 0.00 | 0.55 | 0.07 | 0.00 | 0.84 | 0.10 | 0.00 | 0.42 |
| p_Bacteroidetes  c_Bacteroidia  o_Bacteroidales  f_Prevotellaceae  g_UC Prevotellaceae | 0.79 | 0.99 | 0.64 | 1.00 | 0.10 | 0.00 | 1.97 | 0.06 | 0.00 | 1.94 | 0.07 | 0.00 | 1.68 |
| p_Tenericutes  c_Mollicutes  o_RF9  f_UC RF9  g_UC RF9 | 0.81 | 0.99 | 0.50 | 1.00 | 0.09 | 4.4E-04 | 7.00 | 0.12 | 1.2E-03 | 5.93 | 0.24 | 8.4E-04 | 7.92 |
| p_Firmicutes  c_Clostridia  o_Clostridiales  f_Lachnospiraceae  g_Butyrivibrio | 0.62 | 0.92 | 0.59 | 1.00 | 0.09 | 0.00 | 2.39 | 0.17 | 0.00 | 3.69 | 0.02 | 4.5E-04 | 1.59 |
| p_Firmicutes  c_Clostridia  o_Clostridiales  f_vadinBB60  g_UC vadinBB60 | 0.09 | 0.67 | 0.32 | 1.00 | 0.09 | 0.00 | 0.95 | 0.02 | 0.00 | 0.26 | 0.02 | 0.00 | 1.05 |
| p_Firmicutes  c_Clostridia  o_Clostridiales  f_Ruminococcaceae  g_Anaerotruncus | 0.38 | 0.82 | 0.70 | 1.00 | 0.06 | 0.00 | 1.31 | 0.03 | 0.00 | 1.02 | 0.03 | 0.00 | 0.38 |
| p_Firmicutes  c_Clostridia  o_Clostridiales  f_Clostridiaceae 1  g_UC Clostridiaceae 1 | 0.22 | 0.76 | 0.31 | 1.00 | 0.06 | 1.5E-03 | 0.75 | 0.02 | 0.00 | 0.62 | 0.09 | 0.00 | 0.97 |
| p_Firmicutes  c_Clostridia  o_Clostridiales  f_Lachnospiraceae  g_Dorea | 0.90 | 1.00 | 0.79 | 1.00 | 0.06 | 0.01 | 0.27 | 0.06 | 0.01 | 0.22 | 0.05 | 0.01 | 0.17 |
| p_Firmicutes  c_Bacilli  o_Lactobacillales  f_Streptococcaceae  g_Streptococcus | 0.06 | 0.67 | 0.27 | 1.00 | 0.04 | 0.01 | 5.65 | 0.10 | 0.01 | 6.52 | 0.07 | 3.8E-03 | 2.11 |
| p_Firmicutes  c_Negativicutes  o_Selenomonadales  f_Veillonellaceae  g_Veillonella | 0.58 | 0.91 | 0.50 | 1.00 | 0.04 | 1.3E-03 | 6.48 | 0.04 | 1.5E-03 | 2.13 | 0.05 | 3.9E-03 | 0.95 |
| p_Actinobacteria  c_Coriobacteriia  o_Coriobacteriales  f_Coriobacteriaceae  g_UC Coriobacteriaceae | 0.95 | 1.00 | 0.50 | 1.00 | 0.03 | 0.00 | 0.08 | 0.03 | 4.2E-04 | 0.12 | 0.01 | 0.00 | 0.09 |
| p_Firmicutes  c_Clostridia  o_Clostridiales  f_Defluviitaleaceae  g_UC Defluviitaleaceae | 0.50 | 0.85 | 0.64 | 1.00 | 0.02 | 0.00 | 0.50 | 0.03 | 0.01 | 0.28 | 0.03 | 4.3E-03 | 0.13 |
| p_Firmicutes  c_Clostridia  o_Clostridiales  f_Ruminococcaceae  g_Subdoligranulum | 0.07 | 0.67 | 0.84 | 1.00 | 0.02 | 0.00 | 0.11 | 0.04 | 0.00 | 0.21 | 0.04 | 0.01 | 0.14 |
| p_Firmicutes  c_Clostridia  o_Clostridiales  f_Family XIII  g_UC Family XIII | 0.38 | 0.82 | 0.68 | 1.00 | 0.02 | 0.00 | 0.15 | 0.05 | 0.00 | 0.18 | 0.03 | 0.00 | 0.10 |
| p_Proteobacteria  c_Deltaproteobacteria  o_Desulfovibrionales  f_Desulfovibrionaceae  g_Desulfovibrio | 0.38 | 0.82 | 0.91 | 1.00 | 0.02 | 0.00 | 0.96 | 0.04 | 0.00 | 0.73 | 0.04 | 0.00 | 0.37 |
| p_Bacteroidetes  c_Bacteroidia  o_Bacteroidales  f_Porphyromonadaceae  g_Odoribacter | 0.24 | 0.81 | 0.34 | 1.00 | 0.02 | 0.00 | 0.38 | 0.02 | 3.9E-04 | 0.49 | 0.01 | 0.00 | 0.57 |
| p_Actinobacteria  c_Coriobacteriia  o_Coriobacteriales  f_Coriobacteriaceae  g_Olsenella | 0.41 | 0.83 | 0.54 | 1.00 | 0.01 | 0.00 | 3.94 | 3.9E-03 | 0.00 | 0.93 | 4.6E-03 | 0.00 | 1.37 |
| p_Firmicutes  c_Clostridia  o_Clostridiales  f_Lachnospiraceae  g_Howardella | 0.19 | 0.76 | 0.82 | 1.00 | 0.01 | 0.00 | 0.04 | 0.01 | 0.00 | 0.03 | 0.01 | 0.00 | 0.03 |
| p_Firmicutes  c_UC Firmicutes  o_UC Firmicutes  f_UC Firmicutes  g_UC Firmicutes | 0.36 | 0.82 | 0.79 | 1.00 | 0.01 | 0.00 | 1.95 | 0.00 | 4.0E-04 | 0.67 | 0.01 | 0.00 | 0.25 |
| p_Proteobacteria  c_Gammaproteobacteria  o_Aeromonadales  f_Succinivibrionaceae  g_Succinivibrio | 0.39 | 0.82 | 0.05 | 0.94 | 0.01 | 7.0E-04 | 8.40 | 0.22 | 6.5E-04 | 16.63 | 0.11 | 1.1E-03 | 8.41 |
| p_Firmicutes  c_Erysipelotrichia  o_Erysipelotrichales  f_Erysipelotrichaceae  g_UC Erysipelotrichaceae | 0.17 | 0.73 | 0.27 | 1.00 | 0.01 | 0.00 | 0.25 | 4.5E-03 | 0.00 | 0.18 | 2.1E-03 | 0.00 | 0.06 |
| p_Firmicutes  c_Erysipelotrichia  o_Erysipelotrichales  f_Erysipelotrichaceae  g_Asteroleplasma | 0.47 | 0.85 | 0.83 | 1.00 | 0.01 | 0.00 | 0.49 | 0.01 | 0.00 | 0.99 | 0.02 | 0.00 | 0.68 |
| p_Proteobacteria  c_Gammaproteobacteria  o_B38  f_UC B38  g_UC B38 | 1.00 | 1.00 | 0.06 | 0.97 | 0.01 | 0.00 | 0.44 | 0.01 | 0.00 | 0.22 | 1.9E-03 | 0.00 | 0.03 |
| p_Firmicutes  c_Negativicutes  o_Selenomonadales  f_Veillonellaceae  g_Megamonas | 0.33 | 0.82 | 0.27 | 1.00 | 0.01 | 1.9E-03 | 6.51 | 0.01 | 2.1E-03 | 15.24 | 0.01 | 3.2E-03 | 20.50 |
| p_Firmicutes  c_Negativicutes  o_Selenomonadales  f_Veillonellaceae  g_Allisonella | 0.63 | 0.92 | 0.97 | 1.00 | 0.01 | 0.00 | 0.09 | 0.01 | 0.00 | 0.19 | 0.01 | 0.00 | 0.08 |
| p_Firmicutes  c_Negativicutes  o_Selenomonadales  f_Veillonellaceae  g_UC Veillonellaceae | 0.12 | 0.67 | 0.62 | 1.00 | 0.01 | 0.00 | 0.80 | 5.7E-04 | 0.00 | 0.86 | 0.01 | 0.00 | 0.14 |
| p_Proteobacteria  c_Gammaproteobacteria  o_Enterobacteriales  f_Enterobacteriaceae  g_Pantoea | 1.00 | 1.00 | 0.08 | 0.99 | 0.01 | 0.00 | 0.06 | 0.00 | 0.00 | 0.14 | 6.3E-04 | 0.00 | 0.08 |
| p_Actinobacteria  c_Coriobacteriia  o_Coriobacteriales  f_Coriobacteriaceae  g_Slackia | 0.77 | 0.98 | 1.00 | 1.00 | 0.01 | 0.00 | 0.04 | 0.01 | 0.00 | 0.05 | 0.01 | 0.00 | 0.09 |
| p_Proteobacteria  c_Betaproteobacteria  o_Burkholderiales  f_Comamonadaceae  g_UC Comamonadaceae | 0.68 | 0.94 | 0.36 | 1.00 | 0.01 | 0.00 | 0.18 | 7.1E-04 | 0.00 | 1.53 | 0.00 | 0.00 | 0.58 |
| p_Firmicutes  c_Erysipelotrichia  o_Erysipelotrichales  f_Erysipelotrichaceae  g_Turicibacter | 0.31 | 0.82 | 0.21 | 1.00 | 4.8E-03 | 0.00 | 0.45 | 0.01 | 0.00 | 0.05 | 3.6E-03 | 0.00 | 0.22 |
| p_Proteobacteria  c_Betaproteobacteria  o_Burkholderiales  f_Alcaligenaceae  g_Parasutterella | 0.54 | 0.88 | 0.76 | 1.00 | 4.6E-03 | 0.00 | 0.57 | 2.1E-03 | 0.00 | 0.50 | 9.5E-04 | 0.00 | 0.81 |
| p_Cyanobacteria  c_Melainabacteria  o_Gastranaerophilales  f_UC Gastranaerophilales  g_UC Gastranaerophilales | 0.64 | 0.92 | 0.85 | 1.00 | 4.4E-03 | 0.00 | 1.51 | 1.4E-03 | 0.00 | 0.57 | 3.3E-03 | 0.00 | 4.04 |
| p_Actinobacteria  c_Actinobacteria  o_Micrococcales  f_Micrococcaceae  g_Rothia | 0.67 | 0.94 | 0.54 | 1.00 | 3.8E-03 | 0.00 | 0.02 | 2.6E-03 | 4.0E-04 | 0.03 | 2.0E-03 | 3.5E-04 | 0.02 |
| p_Firmicutes  c_Clostridia  o_Clostridiales  f_Defluviitaleaceae  g_Incertae Sedis | 0.38 | 0.82 | 0.97 | 1.00 | 3.6E-03 | 0.00 | 0.02 | 2.2E-03 | 0.00 | 0.02 | 3.5E-03 | 0.00 | 0.03 |
| p_Firmicutes  c_Clostridia  o_Clostridiales  f_Peptococcaceae  g_Peptococcus | 0.64 | 0.92 | 0.36 | 1.00 | 3.5E-03 | 0.00 | 0.09 | 4.7E-03 | 0.00 | 0.08 | 3.4E-03 | 0.00 | 0.04 |
| p_Firmicutes  c_Clostridia  o_Clostridiales  f_Lachnospiraceae  g_probable genus 10 | 0.06 | 0.67 | 0.68 | 1.00 | 2.9E-03 | 3.7E-04 | 0.02 | 0.01 | 6.4E-04 | 0.04 | 1.9E-03 | 3.9E-04 | 0.09 |
| p_Firmicutes  c_Clostridia  o_Clostridiales  f_Ruminococcaceae  g_Oscillibacter | 0.57 | 0.91 | 0.97 | 1.00 | 2.9E-03 | 0.00 | 0.19 | 0.02 | 0.00 | 0.09 | 0.01 | 0.00 | 0.09 |
| p_Proteobacteria  c_Gammaproteobacteria  o_Enterobacteriales  f_Enterobacteriaceae  g_Raoultella | 0.34 | 0.82 | 0.16 | 1.00 | 2.8E-03 | 0.00 | 0.05 | 1.9E-04 | 0.00 | 0.08 | 0.00 | 0.00 | 0.00 |
| p_Bacteroidetes  c_Bacteroidia  o_Bacteroidales  f_Porphyromonadaceae  g_Barnesiella | 0.41 | 0.83 | 0.91 | 1.00 | 2.6E-03 | 0.00 | 0.31 | 1.3E-03 | 0.00 | 0.51 | 2.1E-03 | 0.00 | 0.59 |
| p_Firmicutes  c_Clostridia  o_Clostridiales  f_Clostridiaceae 1  g_Clostridium sensu stricto 1 | 0.91 | 1.00 | 0.30 | 1.00 | 2.3E-03 | 0.00 | 0.16 | 1.8E-03 | 0.00 | 0.10 | 2.5E-03 | 0.00 | 0.02 |
| p_Bacteroidetes  c_Bacteroidia  o_Bacteroidales  f_Rikenellaceae  g_RC9 gut group | 0.07 | 0.67 | 0.68 | 1.00 | 1.8E-03 | 0.00 | 1.70 | 0.06 | 0.00 | 5.45 | 2.1E-03 | 0.00 | 4.91 |
| p_Firmicutes  c_Clostridia  o_Clostridiales  f_Christensenellaceae  g_Christensenella | 0.65 | 0.93 | 0.94 | 1.00 | 1.6E-03 | 0.00 | 0.01 | 0.00 | 0.00 | 0.03 | 8.4E-04 | 0.00 | 3.2E-03 |
| p_Lentisphaerae  c_Lentisphaeria  o_Victivallales  f_Victivallaceae  g_Victivallis | 0.50 | 0.85 | 0.91 | 1.00 | 1.5E-03 | 0.00 | 0.19 | 1.1E-03 | 0.00 | 0.01 | 0.00 | 0.00 | 0.10 |
| p_Firmicutes  c_Clostridia  o_Clostridiales  f_Lachnospiraceae  g_Coprococcus | 2.4E-03 | 0.28 | 0.57 | 1.00 | 1.4E-03 | 0.00 | 0.01 | 9.4E-04 | 0.00 | 0.01 | 1.8E-03 | 0.00 | 0.01 |
| p_Firmicutes  c_Clostridia  o_Clostridiales  f_Lachnospiraceae  g_Anaerostipes | 0.05 | 0.67 | 0.46 | 1.00 | 1.4E-03 | 0.00 | 0.03 | 1.5E-04 | 0.00 | 2.0E-03 | 0.00 | 0.00 | 0.01 |
| p_Proteobacteria  c_Deltaproteobacteria  o_Desulfovibrionales  f_Desulfovibrionaceae  g_Bilophila | 0.38 | 0.82 | 0.70 | 1.00 | 1.3E-03 | 0.00 | 0.08 | 0.01 | 0.00 | 0.09 | 0.01 | 0.00 | 0.10 |
| p_Firmicutes  c_Negativicutes  o_Selenomonadales  f_Acidaminococcaceae  g_Phascolarctobacterium | 0.33 | 0.82 | 0.59 | 1.00 | 1.2E-03 | 0.00 | 1.56 | 4.4E-03 | 0.00 | 3.24 | 4.6E-03 | 0.00 | 1.15 |
| p_Proteobacteria  c_Gammaproteobacteria  o_Pasteurellales  f_Pasteurellaceae  g_Actinobacillus | 0.92 | 1.00 | 1.00 | 1.00 | 1.2E-03 | 0.00 | 0.01 | 3.2E-04 | 0.00 | 0.03 | 0.00 | 0.00 | 0.08 |
| p_Actinobacteria  c_Coriobacteriia  o_Coriobacteriales  f_Coriobacteriaceae  g_Atopobium | 0.04 | 0.67 | 0.16 | 1.00 | 1.1E-03 | 0.00 | 0.60 | 0.00 | 0.00 | 0.11 | 0.00 | 0.00 | 0.98 |
| p_Firmicutes  c_Bacilli  o_Lactobacillales  f_Leuconostocaceae  g_Weissella | 0.41 | 0.83 | 0.38 | 1.00 | 1.0E-03 | 0.00 | 0.02 | 4.1E-04 | 0.00 | 0.02 | 0.00 | 0.00 | 0.01 |
| p_Verrucomicrobia  c_Verrucomicrobiae  o_Verrucomicrobiales  f_Verrucomicrobiaceae  g_Akkermansia | 0.21 | 0.76 | 0.73 | 1.00 | 8.4E-04 | 0.00 | 1.38 | 1.7E-04 | 0.00 | 0.73 | 0.00 | 0.00 | 0.02 |
| p_Firmicutes  c_Bacilli  o_Lactobacillales  f_Enterococcaceae  g_Enterococcus | 0.76 | 0.98 | 0.50 | 1.00 | 5.7E-04 | 0.00 | 0.01 | 1.7E-04 | 0.00 | 1.12 | 0.00 | 0.00 | 0.02 |
| p_Tenericutes  c_Mollicutes  o_NB1-n  f_UC NB1-n  g_UC NB1-n | 0.19 | 0.76 | 0.50 | 1.00 | 5.6E-04 | 0.00 | 0.29 | 9.2E-04 | 0.00 | 0.20 | 0.00 | 0.00 | 1.80 |
| p_Proteobacteria  c_UC Proteobacteria  o_UC Proteobacteria  f_UC Proteobacteria  g_UC Proteobacteria | 0.03 | 0.67 | 0.01 | 0.77 | 4.4E-04 | 0.00 | 0.04 | 3.0E-03 | 0.00 | 0.08 | 0.00 | 0.00 | 0.09 |
| p_Firmicutes  c_Negativicutes  o_Selenomonadales  f_Veillonellaceae  g_Mitsuokella | 0.08 | 0.67 | 0.38 | 1.00 | 4.1E-04 | 0.00 | 0.03 | 0.00 | 0.00 | 0.01 | 3.8E-04 | 0.00 | 0.11 |
| p_Bacteroidetes  c_Bacteroidia  o_Bacteroidales  f_Porphyromonadaceae  g_Butyricimonas | 0.84 | 1.00 | 0.84 | 1.00 | 4.1E-04 | 0.00 | 0.22 | 0.00 | 0.00 | 0.24 | 0.00 | 0.00 | 0.01 |
| p_Spirochaetae  c_Spirochaetes  o_Spirochaetales  f_Spirochaetaceae  g_Treponema | 0.90 | 1.00 | 0.43 | 1.00 | 3.6E-04 | 0.00 | 0.22 | 3.9E-04 | 0.00 | 0.90 | 0.00 | 0.00 | 6.69 |
| p_Firmicutes  c_Clostridia  o_Clostridiales  f_Ruminococcaceae  g_Flavonifractor | 0.08 | 0.67 | 0.42 | 1.00 | 3.3E-04 | 0.00 | 0.09 | 1.4E-03 | 0.00 | 0.12 | 1.1E-03 | 0.00 | 0.21 |
| p_Firmicutes  c_Erysipelotrichia  o_Erysipelotrichales  f_Erysipelotrichaceae  g_UC _Erysipelotrichaceae | 0.11 | 0.67 | 0.22 | 1.00 | 2.0E-04 | 0.00 | 0.16 | 0.00 | 0.00 | 0.02 | 0.00 | 0.00 | 0.19 |
| p_Firmicutes  c_Clostridia  o_Clostridiales  f_Lachnospiraceae  g_Oribacterium | 0.52 | 0.86 | 0.73 | 1.00 | 2.0E-04 | 0.00 | 0.98 | 4.6E-04 | 0.00 | 1.02 | 1.3E-03 | 0.00 | 1.16 |
| p_Proteobacteria  c_Gammaproteobacteria  o_Enterobacteriales  f_Enterobacteriaceae  g_Hafnia | 0.92 | 1.00 | 0.85 | 1.00 | 2.0E-04 | 0.00 | 0.09 | 3.5E-04 | 0.00 | 0.03 | 0.00 | 0.00 | 0.11 |
| p_Firmicutes  c_Negativicutes  o_Selenomonadales  f_Veillonellaceae  g_Anaerovibrio | 0.97 | 1.00 | 0.49 | 1.00 | 1.7E-04 | 0.00 | 1.10 | 3.1E-04 | 0.00 | 2.97 | 0.00 | 0.00 | 0.33 |
| p_Actinobacteria  c_Coriobacteriia  o_Coriobacteriales  f_Coriobacteriaceae  g_Enterorhabdus | 0.58 | 0.91 | 1.00 | 1.00 | 0.00 | 0.00 | 0.04 | 0.00 | 0.00 | 0.01 | 0.00 | 0.00 | 0.10 |
| p_Bacteroidetes  c_Bacteroidia  o_Bacteroidales  f_Rikenellaceae  g_UC Rikenellaceae | 0.05 | 0.67 | 0.47 | 1.00 | 0.00 | 0.00 | 0.04 | 8.0E-04 | 0.00 | 0.09 | 0.00 | 0.00 | 0.08 |
| p_Bacteroidetes  c_Bacteroidia  o_Bacteroidales  f_Rikenellaceae  g_dgA-11 gut group | 0.50 | 0.85 | 1.00 | 1.00 | 0.00 | 0.00 | 7.9E-04 | 0.00 | 0.00 | 0.01 | 0.00 | 0.00 | 0.11 |
| p_Elusimicrobia  c_Elusimicrobia  o_Elusimicrobiales  f_Elusimicrobiaceae  g_Elusimicrobium | 0.69 | 0.94 | 0.58 | 1.00 | 0.00 | 0.00 | 0.44 | 1.7E-04 | 0.00 | 0.52 | 0.00 | 0.00 | 0.42 |
| p_Firmicutes  c_Clostridia  o_Clostridiales  f_Clostridiaceae 1  g_Sarcina | 1.00 | 1.00 | 0.50 | 1.00 | 0.00 | 0.00 | 0.02 | 0.00 | 0.00 | 3.0E-04 | 0.00 | 0.00 | 0.24 |
| p_Firmicutes  c_Clostridia  o_Clostridiales  f_vadinBB60  g_UC rumen bacterium | 0.22 | 0.76 | 0.03 | 0.77 | 0.00 | 0.00 | 0.06 | 0.00 | 0.00 | 2.6E-03 | 0.00 | 0.00 | 0.07 |
| p_Firmicutes  c_Erysipelotrichia  o_Erysipelotrichales  f_Erysipelotrichaceae  g_Holdemania | 0.43 | 0.83 | 0.73 | 1.00 | 0.00 | 0.00 | 0.02 | 1.6E-04 | 0.00 | 0.02 | 3.9E-04 | 0.00 | 0.05 |
| p_Firmicutes  c_Erysipelotrichia  o_Erysipelotrichales  f_Erysipelotrichaceae  g_Solobacterium | 1.00 | 1.00 | 1.00 | 1.00 | 0.00 | 0.00 | 0.18 | 0.00 | 0.00 | 0.04 | 0.00 | 0.00 | 0.03 |
| p_Fusobacteria  c_Fusobacteriia  o_Fusobacteriales  f_Fusobacteriaceae  g_Fusobacterium | 1.00 | 1.00 | 0.75 | 1.00 | 0.00 | 0.00 | 0.00 | 0.00 | 0.00 | 2.4E-03 | 0.00 | 0.00 | 0.09 |
| p_Proteobacteria  c_Alphaproteobacteria  o_Rhodospirillales  f_Rhodospirillaceae  g_Thalassospira | 1.00 | 1.00 | 0.22 | 1.00 | 0.00 | 0.00 | 1.76 | 0.00 | 0.00 | 0.47 | 0.00 | 0.00 | 0.02 |
| p_Proteobacteria  c_Alphaproteobacteria  o_Rhodospirillales  f_Rhodospirillaceae  g_UC Rhodospirillaceae | 1.00 | 1.00 | 0.20 | 1.00 | 0.00 | 0.00 | 1.25 | 1.6E-04 | 0.00 | 0.12 | 1.6E-03 | 0.00 | 0.65 |
| p_Proteobacteria  c_Alphaproteobacteria  o_UC Alphaproteobacteria  f_UC Alphaproteobacteria  g_UC Alphaproteobacteria | 0.50 | 0.85 | 1.00 | 1.00 | 0.00 | 0.00 | 0.41 | 0.00 | 0.00 | 0.00 | 0.00 | 0.00 | 0.06 |
| p_Proteobacteria  c_Betaproteobacteria  o_Burkholderiales  f_Oxalobacteraceae  g_Oxalobacter | 0.75 | 0.98 | 1.00 | 1.00 | 0.00 | 0.00 | 0.00 | 0.00 | 0.00 | 0.07 | 0.00 | 0.00 | 0.00 |
| p_Proteobacteria  c_Betaproteobacteria  o_Neisseriales  f_Neisseriaceae  g_Neisseria | 0.38 | 0.82 | 0.63 | 1.00 | 0.00 | 0.00 | 0.18 | 0.00 | 0.00 | 1.63 | 0.00 | 0.00 | 1.76 |
| p_Proteobacteria  c_Betaproteobacteria  o_UC Betaproteobacteria  f_UC Betaproteobacteria  g_UC Betaproteobacteria | 1.00 | 1.00 | 1.00 | 1.00 | 0.00 | 0.00 | 0.06 | 1.6E-04 | 0.00 | 0.06 | 0.00 | 0.00 | 0.06 |
| p_Proteobacteria  c_Epsilonproteobacteria  o_Campylobacterales  f_Campylobacteraceae  g_Campylobacter | 0.63 | 0.92 | 0.25 | 1.00 | 0.00 | 0.00 | 0.35 | 0.00 | 0.00 | 0.12 | 0.00 | 0.00 | 0.02 |
| p_Proteobacteria  c_Gammaproteobacteria  o_Aeromonadales  f_Succinivibrionaceae  g_UC Succinivibrionaceae | 0.82 | 1.00 | 0.08 | 0.99 | 0.00 | 0.00 | 0.30 | 4.5E-04 | 0.00 | 0.26 | 6.3E-04 | 0.00 | 0.01 |
| p_Spirochaetae  c_Spirochaetes o_Spirochaetales  f_Brachyspiraceae  g_Brachyspira | 1.00 | 1.00 | 1.00 | 1.00 | 0.00 | 0.00 | 0.01 | 0.00 | 0.00 | 0.04 | 0.00 | 0.00 | 0.34 |
| p_TM7  c_UC TM7  o_UC TM7  f_UC TM7  g_Candidatus Saccharimonas | 0.63 | 0.92 | 0.88 | 1.00 | 0.00 | 0.00 | 0.30 | 0.00 | 0.00 | 0.18 | 0.00 | 0.00 | 0.16 |
| p_Tenericutes  c_Mollicutes  o_Anaeroplasmatales  f_Anaeroplasmataceae  g_Anaeroplasma | 0.84 | 1.00 | 0.84 | 1.00 | 0.00 | 0.00 | 0.03 | 5.8E-04 | 0.00 | 0.02 | 3.9E-04 | 0.00 | 1.11 |
| p_Verrucomicrobia  c_Opitutae  o_vadinHA64  f_UC vadinHA64  g_UC vadinHA64 | 0.22 | 0.76 | 0.44 | 1.00 | 0.00 | 0.00 | 0.09 | 0.00 | 0.00 | 0.01 | 0.00 | 0.00 | 0.33 |

Values where a particular taxonomc group was absent (abundance = 0) are shown in red font.

Values for low abundances are shown using exponential notation, i.e. as E+n, where E (which stands for Exponent) multiplies the preceding number by 10 to the nth power. For example, 5.8E-04 = 5.8 X 10^-4^ = 0.00058
